# Toxicological Relevance of Biogenic Amines in Honey: Dietary Exposure and Integrated Risk Indicators in Algerian and Moroccan Honeys

**DOI:** 10.3390/foods15081411

**Published:** 2026-04-17

**Authors:** Fabio Bruno, Giuseppe Bruschetta, Anthea Miller, Vincenzo Nava, Patrizia Licata

**Affiliations:** 1 Department of Veterinary Sciences, University of Messina, 98168 Messina, Italy; giuseppe.bruschetta@unime.it (G.B.); plicata@unime.it (P.L.); 2 Department of Biomedical and Dental Sciences and of Morphological and Functional Imagines (BIO-MORF), University of Messina, 98122 Messina, Italy

**Keywords:** *Apis mellifera*, Biogenic amines, estimated daily intake, food safety, honey, toxicological risk

## Abstract

Biogenic amines are nitrogenous compounds that may occur in foods through plant metabolism, bee enzymatic activity, or microbial decarboxylation. This study evaluated biogenic amines content in monofloral honeys from Algeria and Morocco, integrating compositional analysis, quality indices, and dietary exposure assessment within a toxicological risk characterization framework. Eight amines were quantified by HPLC-FLD, and Estimated Daily Intake (EDI) was calculated under adult and pediatric low- and high-consumption scenarios. Composite indices, including Total Biogenic Amines (TBA), Biogenic Amine Index (BAI), Vasoactive Amine Load (VAL), Potentiation Index (PI), and Quality Index (QI), were determined. Marked intra- and inter-city variability was observed, particularly for serotonin, tryptamine, and tyramine. Algerian *Euphorbia orientalis* L. samples showed the highest TBA and VAL values. However, histamine concentrations generally remained below 1 mg/kg, and tyramine levels were markedly lower than doses associated with hypertensive effects. Worst-case EDI values were in the order of 10^−3^–10^−4^ mg/kg body weight/day, including high-consumption pediatric scenarios. PI values were low, indicating limited synergistic amplification by diamines. Overall, despite botanical and geographical variability, the analyzed honeys exhibit a wide safety margin and based on the applied screening-level assessment, no immediate risk is indicated under the considered scenarios.

## Simple Summary

Honey is a natural food of animal origin produced by honeybees and is widely consumed for its nutritional and health-promoting properties. At the same time, honey can reflect both botanical and environmental characteristics of the areas where bees forage. Among the compounds naturally present in honey, biogenic amines are of particular interest because they play important biological roles but may also pose toxicological risks when present at elevated levels. By comparing honeys of distinct floral origin and geographic provenance, we evaluated how plant species and local environmental conditions influence biogenic amine composition. In addition, potential dietary exposure was assessed using established toxicological indices, including Estimated Daily Intake and cumulative risk indicators. Our results provide new insights into the biological and toxicological relevance of biogenic amines in honey and support the use of honey as a valuable indicator of environmental quality and food safety. This information is relevant for consumers, beekeepers, and regulatory authorities interested in the quality and safety of bee-derived products.

## 1. Introduction

Honey is a natural food of animal origin produced by honey bees (*Apis mellifera*) through the enzymatic transformation of floral nectar and honeydew [1]. Beyond its long-standing role as a natural sweetener, honey is widely recognized for its antioxidant, anti-inflammatory, antimicrobial, and health-promoting properties, which have supported its use in traditional medicine and modern nutrition alike [1,2]. In recent years, growing consumer interest in functional foods with preventive and health-supporting effects has further highlighted honey as a nutritionally valuable product contributing to both physical and mental well-being [3,4,5]. Honey bees themselves represent a key animal species within agroecosystems, playing an essential role in pollination, biodiversity conservation, and food security [6,7]. At the same time, bees and bee-derived products are increasingly regarded as sensitive bioindicators of environmental quality, as their foraging behavior and metabolic activity reflect the botanical composition, microbial dynamics, and potential contamination of the surrounding environment [8,9]. In the last few years, increasing attention has been directed toward nitrogenous compounds, including free amino acids and biogenic amines, due to their complex biological roles and potential toxicological implications [10]. Biogenic amines are low-molecular-weight organic bases primarily formed through the decarboxylation of amino acids or through metabolic and microbial activity [11]. In biological systems, they are involved in essential physiological processes such as cell growth, gene regulation, neurotransmission, and modulation of immune and vascular functions [12,13]. Honey and other bee-derived products may contain several biogenic amines, including serotonin, tryptamine, histamine, tyramine, cadaverine, putrescine, spermidine, and spermine, whose biological effects can be either beneficial or adverse depending on their concentration, bioavailability, and metabolic clearance [14,15]. Polyamines such as putrescine, spermidine, and spermine are essential regulators of cell physiology and are generally associated with antioxidant, cytoprotective, and anti-aging effects, contributing to cellular homeostasis, proliferation, and stress resistance [16]. These effects are mainly related to the polycationic nature of polyamines, which allows electrostatic interactions with negatively charged macromolecules such as DNA, RNA, phospholipids, and proteins [16,17]. Through these interactions, polyamines contribute to nucleic acid stabilization and regulate transcriptional and translational processes by modulating DNA- and RNA-binding proteins, ribosome assembly, and mRNA translation, thereby supporting protein synthesis, cell cycle progression, and cellular proliferation and differentiation [16,18,19]. Spermidine plays a crucial role in the post-translational activation of the eukaryotic translation initiation factor 5A (eIF5A) via hypusination, a unique modification essential for efficient protein synthesis, cell growth, and autophagy regulation [20]. In addition, spermidine has been linked to the activation of autophagic pathways that contribute to cellular renewal, oxidative stress resistance, and longevity [21]. Spermine, beyond its structural role in nucleic acid stabilization, regulates ion channel activity, membrane permeability, and intracellular calcium homeostasis, which are fundamental for cellular signaling and survival [19]. Putrescine serves as the metabolic precursor of higher polyamines and is generated through ornithine decarboxylation, representing a key regulatory step in polyamine biosynthesis and growth control [22]. Conversely, excessive intake of certain monoamines, particularly histamine and tyramine, is associated with toxicological risks, including headaches, hypertension, gastrointestinal disturbances, and pseudoallergic reactions [14]. From a food safety perspective, cadaverine and putrescine are of particular interest, as they are commonly linked to protein degradation and microbial decarboxylase activity [23]. Although these diamines may participate in physiological processes such as cellular metabolism, pH regulation, membrane stabilization, and tissue remodeling, their presence at elevated levels in foods is generally considered an indicator of poor hygienic quality, fermentation, or degradation processes [23]. Moreover, cadaverine and putrescine may potentiate the toxic effects of other biogenic amines by competing with detoxification pathways, thereby reducing metabolic clearance and enhancing systemic exposure [23]. L-tryptophan (TRP), an essential amino acid present in honey, is the metabolic precursor of serotonin (5-hydroxytryptamine, 5-HT), a biogenic amine with relevant physiological activity [24,25]. Although mainly known for its role in neurotransmission, serotonin acts predominantly in peripheral tissues, regulating vascular and gastrointestinal functions, immune responses, and intracellular signaling. Its presence in food matrices is therefore relevant from a functional and toxicological perspective, particularly due to interactions with other dietary amines [26,27]. The concentration and profile of biogenic amines and TRP-related compounds in honey are influenced by multiple factors, including botanical and geographic origin, bee species, environmental conditions, seasonality, enzymatic activity, microbial load, harvesting practices, and storage conditions [28]. Furthermore, polyamines and related biogenic amines are involved in insect development, immune responses, and stress adaptation, suggesting that their occurrence in honey may reflect both plant-derived precursors and metabolic processes occurring within the bee organism. As a result, honeys from different regions may display distinct biochemical signatures that integrate ecological, biological, and technological factors [29]. In North African countries such as Algeria and Morocco, several studies have focused on the physicochemical characterization and botanical origin of honey, confirming its high nutritional and commercial value. However, to the best of our knowledge, the occurrence and distribution of biogenic amines in honey from this region have received very limited attention. Although biogenic amines have been extensively studied in other food matrices, including fermented and protein-rich products, their presence in honey remains poorly characterized, particularly in relation to geographical and botanical variability [30,31,32]. Therefore, this study aims to address this knowledge gap by providing a comprehensive assessment of biogenic amine profiles in monofloral honeys from Algeria and Morocco, combined with a screening-level evaluation of dietary exposure.

In this context, the present study aimed to investigate honey as a biologically and toxicologically relevant animal-derived food matrix by characterizing the occurrence and distribution of biogenic amines in samples of different botanical origin and geographic provenance collected in Algeria and Morocco. The study focused on the quantitative determination of tryptamine, histamine, tyramine, cadaverine, putrescine, spermidine, spermine, and serotonin, selected for their biological activity, toxicological relevance, and involvement in physiological and degradative processes. By comparing honeys from distinct botanical species and urban areas, this work sought to evaluate the influence of plant origin and local environmental conditions on the biogenic amine profile. In addition, potential dietary exposure and toxicological relevance were assessed through the calculation of Estimated Daily Intake (EDI) and integrative indices, including Total Biogenic Amines (TBA), Biogenic Amine Index (BAI), vasoactive amine load (VAL), and Potentiation Index (PI), allowing a comprehensive evaluation of consumer safety.

## 2. Materials and Methods

### 2.1. Botanical Species and Sampling Area

A total of 84 honey samples from *A. mellifera* were collected in different regions of Algeria and Morocco between April and September 2025. Honey samples were collected from 3 Algerian and 4 Moroccan cities based on availability and production relevance. Although a fully randomized sampling design was not applicable, a stratified approach was adopted to ensure representation of major monofloral types and key production areas. The number of samples per city was proportionally aligned with local honey production capacity and availability, as declared by beekeepers. This approach aimed to capture geographical and botanical variability while maintaining practical feasibility. In Algeria, samples were obtained from Laghouat, a semi-arid region located at an average altitude of approximately 760 m above sea level and characterized by hot summers, with average temperatures ranging from 35 to 38 °C, and mild winters with temperatures between 10 and 15 °C. Honey samples from this area included floral origins of *Eucalyptus globulus*, *Ziziphus lotus*, *Euphorbia orientalis* L., *Tamarix* L., as well as multifloral types. Samples were also collected from Tiaret, situated at about 970 m and characterized by a Mediterranean semi-arid climate. This region experiences summer temperatures of approximately 32–36 °C and winter temperatures ranging from 8 to 12 °C. Honeys from Tiaret included multifloral samples and monofloral honeys derived from *Citrus sinensis*, *Echinops* spp., and *Bunium mauritanicum*. In addition, samples from Tindouf, located in the Saharan desert at an altitude of around 400 m, were included. This area is characterized by extremely arid conditions, with summer temperatures reaching up to 42 °C and cooler winters ranging from 6 to 12 °C. Honeys collected from Tindouf originated mainly from *Euphorbia orientalis* L., *Echinops* spp., *Euphorbia bupleuroides*, and *Peganum harmala* (Table 1).

In Morocco, honey samples were collected from several regions characterized by distinct geographical and climatic conditions. Khénifra, located at an altitude of approximately 890 m, is characterized by a continental climate with warm summers, during which temperatures typically range from 30 to 35 °C, and cold winters with temperatures between 5 and 10 °C. Beni Mellal, situated at an altitude of about 620 m, experiences a semi-arid climate with very hot summers, reaching temperatures of 38–40 °C, and milder winters ranging from 8 to 12 °C. Samples were also obtained from Azilal, a mountainous area within the High Atlas range, located at altitudes between 1200 and 1370 m. This region is characterized by cooler summer temperatures of approximately 25–30 °C and cold winters, during which temperatures may drop to 0–5 °C. Finally, honey samples from Fquih Ben Salah (437 m) were collected from a lowland area with warm and dry conditions, summer temperatures of 35–38 °C and winter temperatures between 10 and 15 °C. The Moroccan honey samples contained a wide range of floral sources, including *Ziziphus christi*, *Citrus sinensis*, *Euphorbia orientalis* L., *Thymus* spp., *Ziziphus lotus*, *Eucalyptus globulus*, *Globularia alypum*, and multifloral honeys (Table 1).

The selection of the sampling areas was designed to include the main honey-producing regions of Algeria and Morocco and to capture a wide range of environmental, climatic, and botanical conditions potentially influencing the formation and accumulation of biogenic amines in honey. The botanical and geographical origin of the honey samples was initially assigned based on information provided by beekeepers and nutritional labeling. The botanical origin of honey samples was further confirmed through pollen spectrum analysis carried out using melissopalynological methodologies [33].

### 2.2. Melissopalynological Analysis

Pollen analysis was performed by initially diluting honey samples in distilled water, followed by centrifugation to concentrate the pollen fraction. The resulting sediment was resuspended in a glycerol-water solution and mounted on microscope slides. Pollen grains were examined under a light microscope, and a minimum of 300 pollen grains per sample were identified and counted to ensure statistical reliability. Five grams of honey were accurately weighed and dissolved in 10 mL of distilled water previously heated at 35 °C and solution was centrifuged at 3500 rpm for 10 min. The supernatant was carefully decanted. A second centrifugation was then performed under the same conditions for 5 min to ensure complete pollen recovery. Then the sediment was transferred into a microscope slide and the pollen grains were identified and quantified by microscopy, and at least 400 pollen grains per sample were counted to determine the relative frequency of each pollen type. The botanical origin of honey samples was classified as monofloral or multifloral according to established melissopalynological criteria. Pollen identification was carried out by comparison with reference pollen slides and standard pollen atlases. The botanical origin of honey samples was determined based on the relative frequency of pollen grains from different plant taxa, classified as predominant (>45%), secondary (16–45%), important minor (3–15%), or minor (<3%). Honey samples were defined as monofloral when a single pollen taxon was present at a predominant level (≥45%), bifloral when two taxa occurred at secondary levels (16–45%) in the absence of a predominant species, and multifloral when three or more pollen taxa were detected at secondary or lower frequencies without a dominant pollen type. The consistency between the declared botanical origin and the experimental pollen-based classification was assessed for all samples to ensure the reliability of the botanical assignment. All honey samples were collected in sterile, food-grade dark glass containers to prevent light-induced degradation and contamination. During sampling and handling, powder-free nitrile gloves were used to minimize external contamination. Samples were transported under refrigerated conditions and subsequently stored at −20 °C until analysis in order to preserve their chemical stability and prevent post-collection microbial activity. Prior to analysis, each honey sample was thoroughly homogenized to ensure sample uniformity. The concentrations of the main biogenic amines, including serotonin, tryptamine, cadaverine, histamine, tyramine, putrescine, spermidine, and spermine, were determined in triplicate for each sample to ensure analytical accuracy, precision, and reproducibility. Botanical identification was reported at the species level when possible; when pollen morphology did not allow reliable species-level identification, the genus level was adopted and indicated using “spp.”.

### 2.3. Extraction and Derivatization Methods

Serotonin, tryptamine, cadaverine, histamine, putrescine, spermidine and spermine were extracted from honey samples using the method reported by Borges et al. (2022) [34] with modifications. Specifically, 2 g of honey was mixed with 10 mL of 0.1 mol/L hydrochloric acid and centrifuged at 4000 g for 10 min at 4 °C [34]. A 1 mL aliquot of the final extract was mixed with 250 μL of 1,7-diaminoheptane (internal standard), 1 mL of saturated sodium bicarbonate solution, 450 μL of 2 M sodium hydroxide solution and 500 μL of dansyl chloride (DCl, 10 g/L). The latter is the derivatizer used for the detection of analytes. After stirring for 1 min, the resulting mixture is placed at 60 °C for 30 min to facilitate derivatization. After this time, the derivatization and extraction processes are completed by adding 1.7 mL of acetonitrile. The mixture is then centrifuged at 4000 rpm for 10 min. Once the two phases have separated, the supernatant is filtered through 0.22 μm filters and stored in the dark (to avoid degradation processes due to the photosensitivity of the dansyl derivative).

### 2.4. HPLC Analysis

Serotonin, tryptamine, cadaverine, tyramine, histamine, putrescine, spermidine and spermine analysis was performed using high performance liquid chromatography HPLC (Shimadzu Italia, Milan, Italy) equipped with an RF-20A fluorimetric detector (FLD, Shimadzu Italia, Milan, Italy) with fixed wavelengths of 320 nm (Ex) and 523 nm (Em), a DGU-20A5R degasser (Shimadzu Italia, Milan, Italy), an LC-20AD pump (Shimadzu Italia, Milan, Italy), a CTO-20A column thermostat (Shimadzu Italia, Milan, Italy). This is one of the most widely used analytical techniques for determining biogenic amines [35]. The column used for this analysis was a SUPELCOSIL TM LC-18 (25 cm × 4.6 mm, 5 μm) thermostatted at 35 °C. Elution of the various components was achieved using an eluent phase consisting of HPLC water (solvent A) and acetonitrile (solvent B) at a constant flow rate of 0.8 mL/min. The total chromatographic run time was 35 min.

### 2.5. Optimization of HPLC Conditions

**Figure 1:**
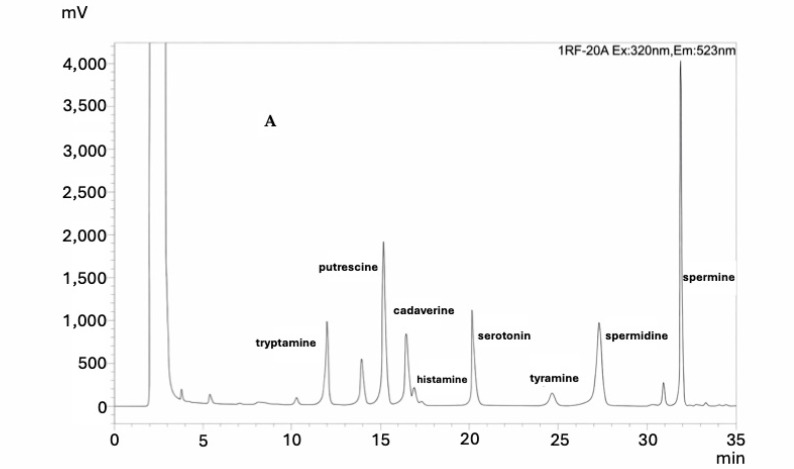

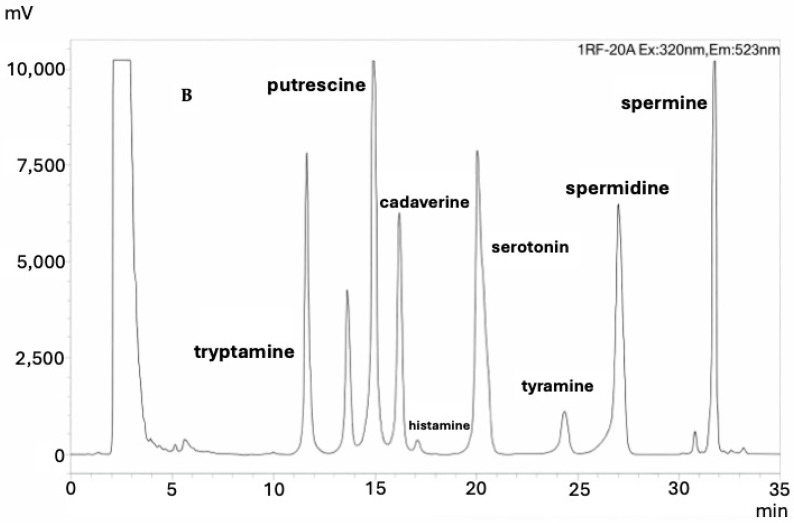
Chromatogram of the 8 biogenic amines. (**A**). Sample; (**B**). Standard.

Several tests were performed to optimise the HPLC-FLD method, varying the following parameters: mobile phase flow, oven temperature and gradient. The type of eluent phase also affects the elution of biogenic amines. In this study, however, the water/acetonitrile mixture was chosen as the eluent phase because it is one of the most widely used mobile phases for analysing biogenic amines, producing results comparable to those obtained using other mixtures [36]. Several trials were performed at 0.6, 0.8 and 1.0 mL/min to investigate the influence of mobile phase flow on retention time. The intermediate value produced the best results. Temperature also plays an important role in peak separation. Therefore, several trials were performed at three different temperatures: 30 °C, 35 °C and 40 °C. The best resolution was achieved at 35 °C. Finally, to improve separation, three gradient elution programmes were designed to separate the eight BAs. However, only one of these programmes, which is reported in Table 2, allowed for the best separation of all biogenic amines in 35 min (Figure 1).

### 2.6. Toxicological Exposure Assessment

Dietary exposure to individual biogenic amines was assessed by estimating the Estimated Daily Intake (EDI) for each compound detected in honey samples. The EDI values were calculated separately for adults and children, considering both average consumption and high-consumption conditions, in order to reflect realistic and conservative patterns of honey intake. Consumption data were derived from established dietary surveys and literature values commonly adopted for exposure assessment of honey and bee products. Body weight reference values were applied to normalize exposure estimates. For the calculation of the EDI, a body weight of 25 kg was assumed for 7-year-old children and a body weight of 70 kg for adults in different consumption scenarios: Adult average consumption (20 g/day; 0.02 kg/day; BW = 70 kg), Adult high consumption (50 g/day; 0.05 kg/day; BW = 70 kg), Child average consumption (10 g/day; 0.01 kg/day; BW = 25 kg), Child high consumption (30 g/day; 0.03 kg/day; BW = 25 kg) [37].The Estimated Daily Intake (EDI) was calculated according to the following equation:

$$EDI=\frac{C\times IR}{BW}$$

EDI is the Estimated Daily Intake (mg/kg body weight/day), C is the mean concentration of the individual biogenic amine (mg/kg), calculated as the average concentration of samples belonging to the same botanical species collected within the same city, IR is the ingestion rate of honey (kg/day) and BW is the body weight (kg). The worst-case scenario was identified as the highest EDI value obtained among the four exposure conditions.

For each biogenic amine, four exposure conditions were considered: adult average consumption, adult high consumption, child average consumption, and child high consumption. This approach allowed evaluation of potential differences in dietary exposure related to age group and consumption habits, and enabled identification of worst-case exposure conditions.

In addition, the Total Biogenic Amines (TBA) content was calculated for each sample as an integrated measure of the overall biogenic amine burden according to the following expression:

$$TBA=\sum_{i=1}^{n}{BA}_{i}$$

TBA is the total biogenic amine content (mg/kg), BA_i_ is the concentration of each quantified biogenic amine (mg/kg), *n* is the number of detected biogenic amines.

TBA was calculated as the sum of all quantified biogenic amines for each botanical species from which honey samples were obtained.

This parameter provides a cumulative estimate of biogenic amine presence and is commonly used as a general indicator of product quality and hygienic status.

To further assess the toxicological relevance of biogenic amine profiles, the Biogenic Amine Index (BAI) was determined. The BAI was used as a composite indicator reflecting the relative contribution of biogenic amines typically associated with microbial activity and product degradation. This index is widely applied in food quality assessment to evaluate freshness and potential spoilage-related risks. The BAI was calculated using the following equation:

BAI=His+Put+Cad+Tyr

BAI is the biogenic amine index (mg/kg), His is the histamine concentration (mg/kg), Tyr is the tyramine concentration (mg/kg), Put is the putrescine concentration (mg/kg) and Cad is the cadaverine concentration (mg/kg). BAI was calculated for each botanical species within the same city as a composite indicator of spoilage-related and microbiologically derived amines.

Moreover, the vasoactive load (VAL) was also estimated as the combined concentration of vasoactive amines according to the following equation:

VAL=His+Tyr

VAL is the vasoactive amine load (mg/kg), His is the histamine concentration (mg/kg) and Tyr is the tyramine concentration (mg/kg). VAL was calculated for each botanical species within the same city to estimate the cumulative concentration of vasoactive amines potentially exerting cardiovascular effects.

This parameter accounts for the combined presence of vasoactive amines, such as histamine and tyramine, which are known to exert physiological effects on the cardiovascular system. The vasoactive load was used to evaluate the potential cumulative vasoactive impact of honey consumption, rather than the effect of single amines in isolation.

Furthermore, a Potentiation Index (PI) was calculated to account for possible synergistic interactions among biogenic amines as follows:

PI = Put + Cad

PI is the potentiation index (dimensionless), Put is the putrescine concentration (mg/kg), Cad is the cadaverine concentration (mg/kg). PI was calculated for each botanical species within the same city to account for the potential enhancing effect of diamines (putrescine and cadaverine) on the biological activity and toxicity of vasoactive amines.

This index considers the ability of putrescine and cadaverine to enhance the biological activity or toxicity of others, particularly through interference with detoxification pathways. The PI was applied as an additional conservative indicator of potential health risk associated with combined exposure to multiple biogenic amines.

A Quality Index (QI) was calculated as a composite parameter expressing the balance between biogenic amines typically associated with quality deterioration and those related to physiological or stabilizing functions, as follows:

$$QI=\frac{Put+Cad+His}{Spd+Spm+1}$$

QI is the quality index (dimensionless), Put is the putrescine concentration (mg/kg), Cad is the cadaverine concentration (mg/kg), His is the histamine concentration (mg/kg), Spd is the spermidine concentration (mg/kg), Spm is the spermine concentration (mg/kg) and 1 is the constant term introduced to ensure numerical stability. QI was calculated for each botanical species within the same city as a normalized ratio between degradation-related amines and physiologically relevant polyamines. Higher QI values indicate a relative prevalence of compounds linked to degradative processes, whereas lower values reflect a biochemical profile consistent with better overall quality.

This multi-parameter approach provided an integrated framework for assessing dietary exposure, quality-related indices, and potential toxicological implications associated with biogenic amines in honey samples.

### 2.7. Statistical Analysis

G Power 3.1 software was used to determine the sample size for the “a priori” ANOVA test (fixed effect, omnibus, one-way), with an effect size (f) of 0.40, a significance level (α) of 0.05, a power (1-β) of 0.80, and twenty-eight groups. Statistical analyses were carried out using GraphPad Prism 9.0 (GraphPad Software, Inc., Boston, MA, USA). Shapiro–Wilk normality was performed, and data were reported as the mean ± SD. Differences among samples were assessed using one-way ANOVA, followed by Bonferroni’s post hoc test. Statistical analyses were conducted considering both geographical origin (city) and botanical classification, in order to evaluate their influence on biogenic amine distribution. Pearson’s correlation analysis was used to assess the relationships among individual biogenic amines, between biogenic amines and the estimated daily intake (EDI), and among EDI values calculated for each amine. Statistical significance was set at *p* < 0.05.

## 3. Results

### 3.1. Method Validation

The analytical method for the determination of serotonin, tryptamine, tyramine, cadaverine, histamine, putrescine, spermidine, and spermine in honey was validated in accordance with Eurachem guidelines and international recommendations for chromatographic methods applied to food matrices [38]. The validation parameters included linearity, sensitivity (limits of detection and quantification), accuracy, and precision.

Linearity was evaluated by constructing calibration curves for each biogenic amine using standard solutions subjected to the same derivatization procedure as the samples. Five concentration levels (range: 10–500 µg/L) were prepared and injected (n = 6). All the standards were prepared using pristine honey extract. Calibration curves were obtained by plotting the peak area ratio of each analyte to the internal standard (1,7-diaminoheptane) versus the corresponding concentration. The linearity of the calibration curves was verified by the correlation coefficients (R^2^). All analytes showed good linearity within the investigated concentration ranges, with coefficients of determination (R^2^) higher than 0.99 for all compounds, indicating an adequate linear response of the HPLC-FLD system.

The limits of detection (LOD) and limits of quantification (LOQ) were reported in Table 2. The LOQs obtained were 0.05 mg/kg for serotonin and tryptamine, and 0.01 mg/kg for cadaverine, tyramine, histamine, putrescine, spermidine, and spermine, confirming the suitability of the method for the determination of biogenic amines at low concentration levels in honey. Accuracy was assessed through recovery experiments performed by spiking honey samples with known amounts of each biogenic amine at two concentration levels representative of low and medium contamination (50 and 500 µg/L). Spiked samples were extracted, derivatized, and analyzed following the same procedure applied to real samples. Recoveries ranged between 91.65 ± 0.65% and 97.20 ± 1.45% for all analytes, demonstrating good accuracy of the method.

At last, the precision of the methods was established. The intraday precision (which means analysing the spiked samples on the same day) and the interday precision (which means evaluating over a longer period of one week) were both assessed. As shown in Table 3, the results, when expressed as relative standard deviation (RSD%), were found to be less than 1.2% and 1.3%, respectively.

### 3.2. Intra-City Assessment of Biogenic Amine Content in Honeys of Identical Botanical Origin

The biogenic amines analyzed serotonin, tryptamine, cadaverine, histamine, tyramine, putrescine, spermidine and spermine were quantified in monofloral and multifloral honey samples of different botanical and geographical origins. The results are reported in Figures S1–S3, which present the mean values ± standard deviation and the statistically significant differences among samples. The intra-city analysis highlights marked quantitative heterogeneity in biogenic amine content among samples belonging to the same geographical area.

In the IDLA4–IDLA6 group, serotonin values of 0.340 ± 0.006 mg/kg (IDLA4), 0.354 ± 0.024 mg/kg (IDLA5), and 0.719 ± 0.028 mg/kg (IDLA6) were observed. Sample IDLA6 was significantly different compared to the previous ones (letters b, c). Cadaverine also showed an increase in IDLA6 (0.110 ± 0.020 mg/kg), significantly higher than IDLA4 and IDLA5 (letters b, c).

In samples IDLA7–IDLA9, serotonin in IDLA9 showed values of 0.159 ± 0.012 mg/kg (letters b, c), higher than IDLA7 and IDLA8, which presented values below the limit of quantification. In samples IDLA7–IDLA9, tyramine showed elevated concentrations. In particular, IDLA8 presented 4.000 ± 1.000 mg/kg (letter a), higher than IDLA7 (1.333 ± 0.577 mg/kg). IDLA9 reported values of 2.333 ± 0.577 mg/kg (letters b, c), higher than IDLA7 but lower than IDLA8. In IDLA8, significant values were also observed for histamine (0.052 ± 0.008 mg/kg; letter a) and putrescine (0.087 ± 0.012 mg/kg; letter a), reduced compared to IDLA7 (0.127 ± 0.015 mg/kg and 0.130 ± 0.010 mg/kg, respectively).

In the IDLA10–IDLA12 group, spermidine in IDLA12 showed a value of 0.234 ± 0.089 mg/kg, significantly different (letter b) from IDLA10 (0.037 ± 0.006 mg/kg).

In samples IDTA13–IDTA15, significant differences were observed for several amines. IDTA13 showed serotonin of 0.236 ± 0.019 mg/kg (letter a), whereas IDTA14 presented values below the limit of quantification; IDTA15 showed 0.287 ± 0.023 mg/kg (letter c). IDTA14 showed tryptamine values of 0.333 ± 0.015 mg/kg (letter a), higher than IDTA13 (0.147 ± 0.006 mg/kg), while IDTA15 presented 0.410 ± 0.030 mg/kg (letter b), higher than IDTA13. IDTA14 showed cadaverine values of 0.133 ± 0.015 mg/kg (letter a), higher than IDTA13 (0.067 ± 0.015 mg/kg). IDTA15 showed 0.127 ± 0.015 mg/kg (letter b), higher than IDTA13. IDTA14 showed putrescine values of 0.027 ± 0.006 mg/kg (letter a), lower than IDTA13 (0.097 ± 0.007 mg/kg). IDTA15 showed 0.147 ± 0.015 mg/kg (letters b, c), higher than IDTA13 and IDTA14. IDTA15 showed spermine values of 0.227 ± 0.015 mg/kg (letters b, c), higher than IDTA13 and IDTA14.

In the IDTA16–IDTA18 group, putrescine in IDTA16 was 0.097 ± 0.015 mg/kg (letter a), higher than IDTA17 (0.047 ± 0.006 mg/kg). IDTA18 showed 0.045 ± 0.003 mg/kg (letter b), lower than IDTA16. Spermidine in IDTA16 (0.017 ± 0.006 mg/kg; letter a) was lower than IDTA17 (0.176 ± 0.055 mg/kg).

In samples IDTA19–IDTA21, serotonin in IDTA20 was 0.419 ± 0.008 mg/kg (letter a), higher than IDTA19 (0.264 ± 0.018 mg/kg). IDTA21 showed 0.429 ± 0.005 mg/kg (letter b), higher than IDTA19. Putrescine in IDTA20 was 0.103 ± 0.015 mg/kg (letter a), higher than IDTA19 (0.046 ± 0.004 mg/kg). Similarly, IDTA21 showed 0.103 ± 0.013 mg/kg (letter b), higher than IDTA19. Spermidine in IDTA21 was 0.280 ± 0.166 mg/kg (letter b), higher than IDTA19.

In the IDTA22–IDTA24 group, cadaverine in IDTA23 was 0.051 ± 0.015 mg/kg (letter a), lower than IDTA22 (0.110 ± 0.020 mg/kg). In IDTA24, cadaverine was 0.071 ± 0.003 mg/kg (letter b), lower than IDTA22. Putrescine in IDTA23 was 0.037 ± 0.012 mg/kg (letter a), lower than IDTA22 (0.106 ± 0.013 mg/kg), while IDTA24 showed 0.038 ± 0.007 mg/kg (letter b), lower than IDTA22.

In samples IDTDA25–IDTDA27, putrescine in IDTDA25 showed values of 0.043 ± 0.004 mg/kg (letters a, b), whereas IDTDA26 and IDTDA27 were below the limit of quantification (<LOQ).

In samples IDTDA28–IDTDA30, serotonin in IDTDA29 was 0.413 ± 0.033 mg/kg (letter a), higher than IDTDA28 (0.284 ± 0.020 mg/kg) and IDTDA30 (0.411 ± 0.030 mg/kg; letter b), both higher than IDTDA28. In IDTDA30, putrescine was 0.053 ± 0.006 mg/kg (letters b, c), whereas IDTDA28 and IDTDA29 were <LOQ.

In the IDTDA31–IDTDA33 group, spermidine in IDTDA32 was 0.257 ± 0.240 mg/kg (letter a), higher than IDTDA31 (0.050 ± 0.017 mg/kg), while IDTDA33 showed 0.439 ± 0.135 mg/kg (letters b, c), higher than IDTDA31 and IDTDA32.

In samples IDKM37–IDKM39, serotonin in IDKM39 was 0.796 ± 0.047 mg/kg (letter c), higher than IDKM38 (0.650 ± 0.048 mg/kg).

In the IDKM40–IDKM42 group, cadaverine in IDKM41 was 0.119 ± 0.009 mg/kg (letter a), higher than IDKM40 (0.060 ± 0.026 mg/kg). IDKM42 showed values of 0.127 ± 0.007 mg/kg (letter b), higher than IDKM40. Putrescine in IDKM41 was 0.104 ± 0.010 mg/kg (letter a), higher than IDKM40 (0.023 ± 0.002 mg/kg). IDKM42 showed values of 0.096 ± 0.007 mg/kg (letter b), slightly higher than IDKM40. Spermine in IDKM41 was 0.123 ± 0.003 mg/kg (letter a) compared to IDKM40 (0.094 ± 0.004 mg/kg). IDKM42 showed values of 0.137 ± 0.005 mg/kg (letter b), higher than IDKM40.

In samples IDKM43–IDKM45, tryptamine in IDKM44 was 0.277 ± 0.012 mg/kg (letter a), lower than IDKM43 (0.287 ± 0.021 mg/kg). IDKM45 showed values of 0.280 ± 0.010 mg/kg (letter b), lower than IDKM43.

In samples IDBM49–IDBM51, tryptamine in IDBM50 was 0.277 ± 0.012 mg/kg (letter a), lower than IDBM49 (0.287 ± 0.021 mg/kg). IDBM51 showed values of 0.280 ± 0.010 mg/kg (letter c) compared to IDBM50.

In samples IDBM55–IDBM57, IDBM56 showed tryptamine values of 0.464 ± 0.013 mg/kg (letter a), higher than IDBM55 (0.423 ± 0.012 mg/kg), while IDBM57 showed 0.287 ± 0.010 mg/kg (letter b), lower than IDBM56.

In samples IDBM58–IDBM60, serotonin in IDBM60 was 0.310 ± 0.012 mg/kg (letter c), higher than IDBM59 (0.173 ± 0.006 mg/kg). Tryptamine in IDBM60 was 0.558 ± 0.008 mg/kg (letter b), higher than IDBM58 (0.374 ± 0.079 mg/kg); furthermore, IDBM60 (letter c) showed higher values than IDBM59 (0.484 ± 0.091 mg/kg).

In samples IDAM61–IDAM63, tryptamine showed significant differences: IDAM62 presented 0.750 ± 0.017 mg/kg (letter a), higher than IDAM61 (0.710 ± 0.040 mg/kg). IDAM63 showed 0.743 ± 0.015 mg/kg (letter b), higher than IDAM61.

Finally, in samples IDFM73–IDFM75, serotonin in IDFM75 reached 0.940 ± 0.084 mg/kg (letter c), lower than IDFM74 (1.112 ± 0.101 mg/kg).

Overall, in Moroccan samples, the most pronounced intra-city differences concerned serotonin and tryptamine.

### 3.3. Inter-City Variability of Biogenic Amines in Honeys of the Same Botanical Origin

Overall, the concentration and distribution of biogenic amines showed significant variability depending on the botanical and geographical origin of the analyzed honeys, as reported in Figures S4–S8.

In *Eucalyptus globulus* honeys, the comparison between Algerian (IDLA1–IDLA3) and Moroccan samples revealed significant differences for several amines.

In Algerian samples, serotonin ranged from 0.159 ± 0.014 to 0.168 ± 0.003 mg/kg, whereas in Moroccan samples it ranged from 0.173 ± 0.006 to 0.317 ± 0.091 mg/kg. Sample IDBM60 (0.310 ± 0.012 mg/kg) showed significant differences compared with Algerian samples (letters a, b), as did IDFM76 and IDFM77 (letters a, b, e).

Tryptamine in Algerian samples ranged from 0.097 ± 0.015 to 0.104 ± 0.008 mg/kg, whereas in Moroccan samples it reached values up to 0.558 ± 0.008 mg/kg (IDBM60), with significant differences compared to IDLA1, IDLA2, and IDLA3 (letters a, b, c). Samples IDFM76–IDFM78 (0.134–0.160 mg/kg) were also significantly different compared to Moroccan samples IDBM58–IDBM60 (letters d, e, f).

For cadaverine, Algerian samples showed concentrations between 0.023 ± 0.003 and 0.027 ± 0.006 mg/kg, whereas Moroccan samples reached up to 0.073 ± 0.006 mg/kg (IDBM58), significantly different from Algerian samples (letters a, b, c). Samples IDFM76 and IDFM78 were also significantly different compared to IDBM58 and IDBM60 (letters d, f).

Tyramine was <LOQ in Algerian samples, whereas it was detectable in Moroccan samples (0.012–0.045 mg/kg). Putrescine was present in both countries, with significant differences between IDFM76 and IDFM77 compared to reference Algerian and Moroccan samples (letters a, b, c).

Overall, for *Eucalyptus globulus*, marked inter-city differentiation was observed, particularly for tryptamine, cadaverine, and serotonin (Figure S4).

In Algerian samples of *Ziziphus lotus* honeys, serotonin ranged from 0.340 ± 0.006 to 0.719 ± 0.028 mg/kg (IDLA6). All Moroccan samples (IDBM55–IDAM66) showed values significantly different from IDLA6 (letter c), with concentrations between 0.307 ± 0.019 and 0.373 ± 0.010 mg/kg.

Tryptamine in Algerian samples ranged from 0.120 ± 0.010 to 0.157 ± 0.025 mg/kg, whereas Moroccan samples ranged from 0.287 ± 0.010 to 0.464 ± 0.013 mg/kg, with significant differences compared to IDLA4, IDLA5, and IDLA6 (letters a, b, c). Furthermore, some samples (IDAM64–IDAM66) were significantly different compared to IDBM56 (letter d).

For cadaverine, Algerian samples showed values up to 0.110 ± 0.020 mg/kg (IDLA6), whereas Moroccan samples ranged from 0.056 ± 0.005 to 0.071 ± 0.003 mg/kg, significantly different from IDLA6 (letter c).

Tyramine was <LOQ in Algerian samples, whereas it was detectable in Moroccan samples (0.027–0.051 mg/kg). Putrescine in Moroccan samples (IDBM55–IDBM57) showed significant differences compared to reference Algerian samples (letters a, b, c) (Figure S5).

In *Euphorbia orientalis* honeys, the occurrence of biogenic amines showed the widest inter-city variability among samples.

Serotonin was <LOQ in IDLA7 and IDLA8, whereas in Moroccan samples it reached up to 0.492 ± 0.091 mg/kg (IDAM71), with highly variable significant differences among samples.

Tryptamine in Algerian samples IDLA7–IDLA9 ranged from 0.140 ± 0.017 to 0.160 ± 0.010 mg/kg, whereas in samples IDTDA25–IDTDA27 it reached 0.588–0.594 mg/kg, with significant differences (letters a, b, c). Moroccan samples ranged between 0.243 ± 0.009 and 0.593 ± 0.015 mg/kg, showing numerous significant differences compared to Algerian samples and among Moroccan groups (letters from a to n).

In Algerian samples IDLA7–IDLA9, tyramine reached high values (1.333 ± 0.577; 4.000 ± 1.000; 2.333 ± 0.577 mg/kg), whereas in Moroccan samples it was <LOQ or ≤0.058 mg/kg. All Moroccan samples showed significant differences compared to Algerian samples (letters a, b, c).

Histamine and putrescine also showed multiple significant differences between Algerian and Moroccan samples, as indicated by letters from a to l (Figure S6).

In multifloral honeys, all biogenic amines were detected in most samples, with wide variability in the levels of serotonin, tryptamine, and putrescine. In Algerian samples, serotonin ranged from <LOQ (IDTA14) to 0.287 ± 0.023 mg/kg (IDTA15). Moroccan samples showed significantly higher values, up to 1.112 ± 0.101 mg/kg (IDFM74), significantly different from IDTA13, IDTA14, and IDTA15 (letters from a to i).

Tryptamine in Algerian samples ranged from 0.147 ± 0.006 to 0.410 ± 0.030 mg/kg, whereas Moroccan samples reached 0.563 ± 0.015 mg/kg (IDFM75), with multiple significant differences compared to Algerian samples (letters from a to i).

Cadaverine and putrescine showed significant differences compared to IDTA13–IDTA15 (letters from a to i). Spermine in Moroccan samples reached up to 0.267 ± 0.012 mg/kg (IDFM73 and IDFM75), significantly different from Algerian samples (letters from a to i) (Figure S7).

In Algerian samples of *Citrus sinensis* honeys, serotonin ranged from 0.428 ± 0.009 to 0.446 ± 0.033 mg/kg. In Moroccan samples, it ranged from 0.189 ± 0.019 to 0.241 ± 0.025 mg/kg, with significant differences compared to IDTA16-IDTA18 (letters a, b, c).

Tryptamine in Algerian samples was stable around 0.370 mg/kg, whereas in Moroccan samples it ranged from 0.277 ± 0.012 to 0.287 ± 0.021 mg/kg; IDKM44 and IDBM50 were significantly different from IDTA16 (letter a).

Cadaverine was detectable in Algerian samples (0.069–0.074 mg/kg), whereas it was <LOQ in all Moroccan samples, with significant differences (letters a, b, c).

Putrescine (letter a), spermidine (letter b), and spermine (letters a, b, c) also showed significant differences compared to reference Algerian samples (Figure S8).

### 3.4. Worst-Case Estimated Daily Intake and Composite Quality Indicators of Biogenic Amines

The theoretical dietary exposure to biogenic amines was calculated under worst-case conditions, considering the maximum concentrations detected among all honey samples analyzed in Algeria and Morocco and applying four distinct consumption scenarios (adult and child; average and high consumption) (Table 4).

Overall, for all analyzed amines, the highest Estimated Daily Intake (EDI) values were observed in the “child—high consumption” scenario, whereas the lowest values systematically corresponded to the “adult—average consumption” scenario.

For serotonin, the EDI calculated for *Ziziphus lotus* (Algeria) ranged from 0.000135 mg/kg bw/day (adult-average consumption) to 0.000566 mg/kg bw/day (child–high consumption). Higher values were estimated for Moroccan multifloral honey, ranging from 0.000294 to 0.001237 mg/kg bw/day, representing the maximum value observed for this amine.

For tryptamine, honey from *Euphorbia orientalis* L. (Algeria) showed an exposure range between 0.000169 and 0.000710 mg/kg bw/day, whereas Moroccan samples of Thymus spp. showed higher values, ranging from 0.000210 to 0.000881 mg/kg bw/day depending on the scenario considered.

Cadaverine EDI remained low in both geographical areas. In honey from *Tamarix* L. (Algeria), it ranged from 0.000040 to 0.000168 mg/kg bw/day, while in Moroccan multifloral honey it ranged from 0.000042 to 0.000178 mg/kg bw/day, showing substantially overlapping values.

For histamine, estimated values for *Euphorbia orientalis* L. were nearly identical between Algeria (0.000058–0.000245 mg/kg bw/day) and Morocco (0.000059–0.000248 mg/kg bw/day).

Tyramine showed the highest exposure values in the entire dataset. In *Euphorbia orientalis* L. honey (Algeria), EDI ranged from 0.000730 mg/kg bw/day (adult-average) to 0.003067 mg/kg bw/day (child-high). In Moroccan multifloral samples, values were markedly lower (0.000028–0.000117 mg/kg bw/day).

For putrescine, EDI in *Euphorbia orientalis* L. (Algeria) ranged from 0.000031 to 0.000128 mg/kg bw/day, while in Moroccan multifloral samples it ranged from 0.000028 to 0.000116 mg/kg bw/day.

Spermidine ranged from 0.000071 to 0.000299 mg/kg bw/day in *Euphorbia bupleuroides* (Algeria), whereas Moroccan multifloral samples showed lower values (0.000026–0.000111 mg/kg bw/day).

For spermine, estimated exposure in *Bunium mauritanicum* (Algeria) ranged from 0.000085 to 0.000358 mg/kg bw/day, whereas in Moroccan multifloral honey it ranged from 0.000076 to 0.000319 mg/kg bw/day.

Overall, even under high-consumption child scenarios, all EDI values were in the order of 10^−3^–10^−4^ mg/kg bw/day, indicating very low exposure levels.

Parallel to the exposure assessment, composite quality indicators were calculated, including Total Biogenic Amines (TBA), the Biogenic Amine Index (BAI), and the Quality Index (QI) (Table 5).

In Algerian samples, TBA ranged from 0.348 mg/kg in *Eucalyptus globulus* to 3.005 mg/kg in *Euphorbia orientalis* L., representing the highest value observed. Most other botanical species showed values between 1.068 and 1.585 mg/kg. The BAI showed marked variability, with a maximum value of 2.800 in *Euphorbia orientalis* L., whereas in the other species it ranged between 0.082 and 0.480. The QI ranged from 0.065 (*Peganum harmala*) to 0.260 (*Euphorbia orientalis* L.), with most samples between 0.111 and 0.237.

In Moroccan samples, TBA was generally lower than the maximum values observed in Algeria, ranging from 0.612 mg/kg (*Eucalyptus globulus*) to 2.348 mg/kg (multifloral). The BAI ranged between 0.064 (*Thymus* spp.) and 0.417 (multifloral), while the QI reached a maximum value of 0.321 in *Euphorbia orientalis* L. In most Moroccan botanical species, QI values ranged between 0.107 and 0.235.

Overall, analysis of the composite parameters highlighted wide variability among botanical species and between countries, with maximum TBA and BAI values observed in Algerian samples of *Euphorbia orientalis* L., whereas the highest TBA value in Morocco was observed in multifloral honey.

The Vasoactive Amine Load (VAL) and Potentiation Index (PI) showed marked variability according to botanical origin and country of production (Table 6).

In Algerian honeys, VAL values ranged from 0.044 mg/kg in *Eucalyptus globulus* to 2.635 mg/kg in *Euphorbia orientalis* L., the latter representing the highest vasoactive burden observed in the entire dataset. Most other Algerian monofloral honeys exhibited relatively low VAL values (<0.35 mg/kg), including *Ziziphus lotus* (0.046 mg/kg), *Citrus sinensis* (0.136 mg/kg), and *Peganum harmala* (0.175 mg/kg). Multifloral samples showed intermediate values (0.174 mg/kg).

The PI in Algerian honeys ranged from 0.032 (*Peganum harmala*) to 0.233 (*Tamarix* L.), indicating variability in the relative contribution of diamines to the vasoactive fraction. Notably, despite the extremely high VAL observed in one *Euphorbia orientalis* L. sample, the corresponding PI (0.165) remained within the overall range observed for Algerian honeys.

In Moroccan samples, VAL values were generally lower and more homogeneous, ranging from 0.001 mg/kg in *Thymus* spp. to 0.253 mg/kg in *Ziziphus christi*. Most samples exhibited VAL values below 0.25 mg/kg, including *Citrus sinensis* (0.102 mg/kg), *Eucalyptus globulus* (0.074–0.108 mg/kg), and *Globularia alypum* (0.140 mg/kg).

The PI in Moroccan honeys ranged from 0.032 (*Ziziphus christi*) to 0.197 (multifloral samples), with the majority of values remaining below 0.15. Overall, Moroccan honeys displayed lower dispersion in both VAL and PI compared to Algerian samples.

### 3.5. Biogenic Amines Correlations in Algeria and Morocco

Correlation analysis between the concentrations of the same and different biogenic amines detected in Algerian (A) and Moroccan (M) honeys revealed several statistically significant associations (Figure S9).

Serotonin A showed positive correlations with cadaverine M (r = 0.259; *p* = 0.0497) and spermidine M (r = 0.462; *p* = 0.0351).

Serotonin M showed significant positive correlations with cadaverine M (r = 0.285; *p* = 0.0147), histamine M (r = 0.261; *p* = 0.021), tyramine M (r = 0.610; *p* ≤ 0.00001), putrescine A (r = −0.531; *p* = 0.0030), putrescine M (r = 0.364; *p* = 0.0129), spermidine M (r = 0.577; *p* = 0.0002), and spermine M (r = 0.763; *p* ≤ 0.00001). It was also negatively correlated with histamine A (r = −0.371; *p* = 0.0021) and tryptamine A (r = −0.311; *p* = 0.0093).

Tryptamine A showed a strong positive correlation with tryptamine M (r = 0.842; *p* ≤ 0.00001), as well as positive associations with cadaverine M (r = 0.402; *p* = 0.0023), histamine A (r = 0.772; *p* ≤ 0.00001), and histamine M (r = 0.459; *p* ≤ 0.0001). It showed negative correlations with tyramine A (r = −0.597; *p* = 0.0043), tyramine M (r = −0.432; *p* = 0.0136), and spermine M (r = −0.784; *p* ≤ 0.00001), and a positive correlation with spermine A (r = 0.423; *p* = 0.0397).

Tryptamine M was positively correlated with cadaverine A (r = 0.332; *p* = 0.0047), cadaverine M (r = 0.473; *p* ≤ 0.0001), histamine A (r = 0.761; *p* ≤ 0.00001), and histamine M (r = 0.577; *p* ≤ 0.00001).

Cadaverine A showed positive correlations with cadaverine M (r = 0.292; *p* = 0.0260), histamine A (r = 0.284; *p* = 0.0210), histamine M (r = 0.362; *p* = 0.0029), tyramine M (r = − 0.352; *p* = 0.0484), and putrescine A (r = 0.522; *p* = 0.0036).

Cadaverine M showed numerous significant positive correlations, particularly with histamine A (r = 0.507; *p* ≤ 0.0001), histamine M (r = 0.574; *p* ≤ 0.00001), tyramine A (r = 0.837; *p* = 0.0001), tyramine M (r = 0.548; *p* = 0.0006), putrescine M (r = 0.625; *p* < 0.0001), spermidine A (r = 0.504; *p* = 0.0199), spermidine M (r = 0.488; *p* = 0.0040), and spermine M (r = 0.447; *p* = 0.0090).

Tyramine A was strongly correlated with spermine A (r = 0.970; *p* ≤ 0.00001) and positively correlated with spermine M (r = 0.531; *p* = 0.0234).

Finally, spermidine M showed a very strong positive correlation with spermine M (r = 0.934; *p* ≤ 0.00001), while putrescine M was positively correlated with spermidine M (r = 0.795; *p* ≤ 0.00001) and spermine M (r = 0.799; *p* ≤ 0.00001).

#### 3.5.1. Correlations Between Biogenic Amines and Exposure Scenarios in Algeria

In Algerian honeys, correlation analysis revealed a limited number of statistically significant associations between biogenic amine concentrations and the estimated exposure scenarios (Figure S9).

In particular, tyramine showed a strong positive correlation with all consumption scenarios considered. It was significantly associated with the Adult–Medium scenario (r = 0.884; *p* ≤ 0.0001), Adult-High (r = 0.885; *p* ≤ 0.0001), Child-Medium (r = 0.884; *p* ≤ 0.0001), and Child-High (r = 0.884; *p* ≤ 0.0001). All coefficients, exceeding 0.88, indicate a very strong and consistent linear relationship between tyramine concentration and the increase in estimated exposure across different population groups.

No additional significant correlations emerged between the other Algerian biogenic amines and the exposure scenarios (*p* > 0.05), suggesting that, in the Algerian context, the variability of estimated exposure is predominantly driven by tyramine concentration.

#### 3.5.2. Correlations Between Biogenic Amines and Exposure Scenarios in Morocco

In contrast to what was observed in Algerian samples, Moroccan honeys exhibited several statistically significant correlations between biogenic amines and exposure scenarios (Figure S9).

Serotonin showed a very strong positive correlation with all considered scenarios, being significantly associated with Adult-Medium (r = 0.888; *p* ≤ 0.0001), Adult-High (r = 0.892; *p* ≤ 0.0001), Child-Medium (r = 0.892; *p* ≤ 0.0001), and Child-High (r = 0.891; *p* ≤ 0.0001). The r values, close to 0.90, indicate an extremely consistent linear relationship between serotonin concentration and increasing estimated exposure under different consumption scenarios.

Tyramine also demonstrated significant positive correlations with all Moroccan scenarios, showing associations with Adult-Medium (r = 0.602; *p* = 0.0135), Adult-High (r = 0.609; *p* = 0.0123), Child-Medium (r = 0.608; *p* = 0.0125), and Child-High (r = 0.609; *p* = 0.0123). Although these coefficients are lower than those observed for serotonin, they still indicate a moderate and statistically robust linear relationship.

Putrescine showed significant positive correlations with Adult-Medium (r = 0.534; *p* = 0.0331), Adult-High (r = 0.539; *p* = 0.0312), Child-Medium (r = 0.539; *p* = 0.0311), and Child-High (r = 0.538; *p* = 0.0315), highlighting a moderate linear association with increasing estimated exposure.

Similarly, spermidine was significantly correlated with Adult-Medium (r = 0.716; *p* = 0.0018), Adult-High (r = 0.721; *p* = 0.0016), Child-Medium (r = 0.719; *p* = 0.0017), and Child-High (r = 0.721; *p* = 0.0016), showing a strong and consistent linear relationship across all scenarios considered.

Finally, spermine also exhibited significant positive correlations with Adult-Medium (r = 0.791; *p* = 0.0003), Adult-High (r = 0.798; *p* = 0.0002), Child-Medium (r = 0.796; *p* = 0.0002), and Child-High (r = 0.798; *p* = 0.0002), with coefficients indicative of a strong linear association between amine concentration and increasing estimated exposure.

Overall, in Moroccan samples, estimated exposure was significantly associated with a greater number of amines compared to Algerian samples, suggesting a stronger influence of the overall amine profile on the variability of estimated intake across different population groups.

#### 3.5.3. Correlations Between Total EDI in Algeria and Morocco

The analysis of correlations between total exposure (EDI) scenarios revealed a markedly distinct behaviour within each country compared to cross-country associations between Algeria and Morocco (Figure S9).

No statistically significant correlations were observed between total exposure scenarios in Algeria and those in Morocco (*p* > 0.05 in all cross-comparisons). Although correlation coefficients between Algerian and Moroccan scenarios were moderately negative (r ranging from −0.528 to −0.542), these associations did not reach statistical significance and therefore cannot be considered indicative of a linear relationship between total exposure levels in the two countries.

## 4. Discussion

The present study provides a comprehensive evaluation of biogenic amine content in monofloral honeys from Algeria and Morocco, integrating compositional data, quality indices, and dietary exposure assessment within a coherent toxicological risk framework. Biogenic amines are low-molecular-weight nitrogenous compounds primarily formed through the enzymatic decarboxylation of free amino acids by microbial decarboxylases, although they may also originate from endogenous plant or animal metabolic pathways [39]. Their presence in honey may reflect multiple sources, including the secondary metabolism of nectar-producing plants, the enzymatic activity of bees, and the potential contribution of microbiota associated with nectar and the hive environment [40]. The marked intra- and inter-city variability observed among the analyzed samples suggests that pedoclimatic, botanical, and microbiological factors influence the final amine profile.

From a biochemical perspective, biogenic amines are mainly generated through the removal of the carboxyl group from specific amino acids: histidine gives rise to histamine, tyrosine to tyramine, ornithine to putrescine, while spermidine and spermine derive from subsequent transformations of putrescine through the addition of aminopropyl groups [13]. In protein-rich matrices such as fish, meat, and aged cheeses, their formation is often associated with fermentation processes or microbial contamination [39,41]. In honey, however, production is generally limited by low water activity, high osmotic pressure, and acidic pH, conditions that inhibit bacterial proliferation and therefore extensive amino acid decarboxylation [40].

From a toxicological standpoint, histamine and tyramine represent the principal vasoactive monoamines among biogenic amines [42]. Histamine may induce vasodilation and allergy-like symptoms at high doses, whereas tyramine exerts a sympathomimetic effect with a potential increase in blood pressure [42]. The clinical relevance of these effects depends on the ingested dose and the efficiency of enzymatic systems (diamine oxidase and monoamine oxidase) in limiting systemic absorption. In the analyzed honeys, the observed concentrations were several orders of magnitude lower than levels associated with documented clinical effects, suggesting a wide safety margin [42].

Physiological polyamines, namely putrescine, spermidine, and spermine, exhibit a different biological profile. These are ubiquitous metabolites present in all eukaryotic cells and are involved in nucleic acid stabilization, modulation of gene expression, and regulation of cell proliferation [43]. The human body synthesizes significant quantities from ornithine and methionine, and dietary intake represents only a fraction of the total body pool [43]. At usual dietary levels, their toxicity is considered negligible; conversely, in some experimental contexts, spermidine has been associated with cytoprotective effects and activation of autophagic mechanisms [43]. In the examined honeys, the detected concentrations were modest and contributed marginally to the overall dietary polyamine load.

Overall, the integration of biochemical knowledge regarding amine formation, understanding of their mechanisms of action, and quantitative exposure assessment allows interpretation of the findings within an evidence-based food safety perspective. Although the presence of biogenic amines represents a useful indicator of botanical and environmental characteristics, the levels observed in Algerian and Moroccan honeys remain compatible with a low-risk profile, confirming that the matrix, due to its intrinsic properties, does not favor toxicologically relevant accumulation of these compounds.

Histamine represents the primary toxicological reference compound in food safety with respect to biogenic amines. In the European Union, Regulation (EC) No 2073/2005 establishes limits between 100 and 200 mg/kg for certain fishery products, thresholds associated with the prevention of scombroid syndrome [44]. No specific regulatory limit exists for honey, likely due to its low susceptibility to microbial proliferation and its low protein content.

In the present study, histamine concentrations detected in *Citrus sinensis*, *Eucalyptus globulus*, and *Euphorbia orientalis* L. samples were below the limits established for fish, generally remaining under 1 mg/kg. Even under a high-consumption pediatric scenario, the calculated Estimated Daily Intake (EDI) was in the order of 10^−4^ mg/kg bw/day or lower. When compared with acute doses associated with clinical manifestations (typically > 0.5–1 mg/kg bw in a single exposure), a wide safety margin emerges. It is also relevant that orally ingested histamine undergoes inactivation by intestinal diamine oxidase; systemic effects mainly occur when this system is saturated or impaired [45]. The levels observed in honey are clearly below those required to saturate physiological metabolic mechanisms.

Tyramine is toxicologically relevant due to its ability to induce catecholamine release and hypertensive crises in individuals treated with monoamine oxidase inhibitors. In the literature, an acute intake of approximately 6–10 mg in a single meal may provoke clinical effects in susceptible individuals [46]. In Algerian samples of *Euphorbia orientalis* L., the highest values were recorded, approaching 4 mg/kg. However, when translated into the consumption scenarios adopted in this study, the estimated intake even in the high-consumption child scenario remains in the order of 10^−3^ mg/kg bw/day, corresponding to absolute amounts markedly lower than the 6 mg per meal threshold. Direct comparison indicates that honey consumption, even in samples with the highest observed concentrations, is unlikely to result in clinically relevant acute exposure.

Serotonin and tryptamine, derived from tryptophan metabolism, are not subject to specific regulatory limits in food. The concentrations observed in Moroccan samples, sometimes higher than those in Algerian samples, suggest a predominantly vegetal rather than microbial origin.

Putrescine, spermidine, and spermine are physiological polyamines naturally present in foods and involved in normal cellular metabolism. Within the Mediterranean diet, total daily polyamine intake may reach several tens of milligrams, with major contributions from cereals, legumes, fermented products, and honey [46]. The levels observed in the present study, even in co-presence of histamine, fall within ranges compatible with normal dietary exposure.

From a toxicological perspective, the geographical and environmental differences between Algeria and Morocco, and the resulting variations in *Citrus sinensis* and *Eucalyptus globulus* honeys, are primarily preventive and interpretative in significance rather than indicative of immediate risk. Despite statistically significant differences in biogenic amine levels between the two countries, the absolute values generally remain well below known toxicological thresholds for histamine, tyramine, and other vasoactive amines. This indicates that even peak values related to climatic or pedological factors do not translate into significant health risks for adults or children. Nevertheless, these differences highlight that amine content may be influenced by exogenous factors modulating plant metabolism and bee enzymatic activity, thereby affecting potential dietary exposure. From a toxicological risk standpoint, this justifies the adoption of conservative scenarios (high consumption, worst-case) and the calculation of integrative indices such as VAL and PI, which consider cumulative or potentiated effects of amines. In summary, geographical and environmental variability does not imply an immediate health hazard but emphasizes the importance of considering production context in exposure estimation and toxicological risk characterization, particularly for foods such as honey in which amine content may vary even among samples of the same botanical origin.

Moreover, considering that honey is produced by *Apis mellifera*, differences in colony physiology and associated microbiota may plausibly contribute to the observed amine profile [47].

Regarding risk characterization, this process integrates hazard identification, dose–response assessment, and estimated exposure. In the case of biogenic amines in honey, the hazard is well defined for histamine and tyramine, whereas no specific toxicological reference thresholds are established for polyamines. Overall, risk characterization indicates that consumption of the analyzed honeys does not pose an appreciable toxicological risk in relation to biogenic amine content. The data support a robust safety profile consistent with the intrinsic properties of the matrix and the absence of significant fermentative phenomena.

Analysis of the Vasoactive Amine Load (VAL) highlights substantial heterogeneity among botanical species and between the two countries considered. In particular, Algerian samples showed an extremely high value in *Euphorbia orientalis* L. (2.635 mg/kg), clearly exceeding other analyzed samples. This finding suggests possible botanical specificity or microenvironmental conditions favoring the formation or preservation of vasoactive amines. In contrast, Moroccan samples generally displayed lower VAL values distributed within a narrower range, with a maximum of 0.253 mg/kg (*Ziziphus christi*). This reduced variability may reflect differences in beekeeping practices, climatic conditions, or phytochemical profiles of floral matrices.

With regard to the Potentiation Index (PI), values were overall low in both countries, suggesting a limited capacity of diamines (putrescine and cadaverine) to amplify the biological activity of vasoactive amines. However, some Algerian samples (*Tamarix* L.) showed relatively higher values, indicating a greater potential for synergistic interaction among different amine classes. Overall, despite some differences related to botanical origin, the observed VAL and PI values remain generally low, supporting a limited toxicological risk under ordinary consumption conditions. Nevertheless, the identification of specific matrices with higher loads underscores the importance of considering botanical origin in integrated toxicological risk of biogenic amines in honey.

However, the overall toxicological assessment indicates that, despite intra- and inter-city variability and concentration peaks observed in certain samples, the levels of biogenic amines detected do not constitute a significant health risk for either adult or pediatric populations. This conclusion also applies under conservative high-consumption scenarios simulating above-average daily intakes and considering the combined presence of vasoactive and potentially synergistic amines. Dietary exposure estimates, integrated with parameters such as Vasoactive Amine Load and Potentiation Index, confirm that measured concentrations remain well below known toxicological thresholds, ensuring a wide safety margin and supporting the absence of acute or chronic adverse effects associated with consumption of these honeys.

## 5. Conclusions

This study provides an integrated biochemical and toxicological evaluation of biogenic amines in monofloral honeys from Algeria and Morocco, combining analytical determination with composite quality indices (TBA, BAI, VAL, PI, QI) and a screening-level dietary exposure assessment. The results revealed a marked variability in BA profiles associated with botanical and geographical origin, with serotonin, tryptamine, and tyramine showing the highest fluctuations. Despite this variability, histamine and tyramine concentrations remained well below levels associated with adverse health effects, and Estimated Daily Intake values were consistently low, even under conservative scenarios, based on a screening-level risk assessment approach. Potentiation Index values further indicated limited synergistic effects among amines. Overall, these findings suggest that, under the investigated conditions, honey does not represent a significant source of exposure to biogenic amines. Beyond this aspect, the study provides novel data for North African monofloral honeys and highlights the usefulness of integrative indicators, such as Vasoactive Amine Load and Potentiation Index, for a more comprehensive interpretation of cumulative exposure. These results contribute to establishing a reference dataset for this region and support future research aimed at refining exposure assessment models and exploring the potential of biogenic amines as indicators of honey quality and origin.

## Figures and Tables

**Table 1:** Sampling locations, botanical origin, and identification codes of honey samples from Algeria and Morocco.

Algeria		
City	Botanical Species	ID
Laghouat	*Eucalyptus globulus*	IDLA1
Laghouat	*Eucalyptus globulus*	IDLA2
Laghouat	*Eucalyptus globulus*	IDLA3
Laghouat	*Ziziphus lotus*	IDLA4
Laghouat	*Ziziphus lotus*	IDLA5
Laghouat	*Ziziphus lotus*	IDLA6
Laghouat	*Euphorbia orientalis* L.	IDLA7
Laghouat	*Euphorbia orientalis* L.	IDLA8
Laghouat	*Euphorbia orientalis* L.	IDLA9
Laghouat	*Tamarix* L.	IDLA10
Laghouat	*Tamarix* L.	IDLA11
Laghouat	*Tamarix* L.	IDLA12
Tiaret	*Multifloral*	IDTA13
Tiaret	*Multifloral*	IDTA14
Tiaret	*Multifloral*	IDTA15
Tiaret	*Citrus sinensis*	IDTA16
Tiaret	*Citrus sinensis*	IDTA17
Tiaret	*Citrus sinensis*	IDTA18
Tiaret	*Echinops* spp.	IDTA19
Tiaret	*Echinops* spp.	IDTA20
Tiaret	*Echinops* spp.	IDTA21
Tiaret	*Bunium mauritanicum*	IDTA22
Tiaret	*Bunium mauritanicum*	IDTA23
Tiaret	*Bunium mauritanicum*	IDTA24
Tindouf	*Euphorbia orientalis* L.	IDTDA25
Tindouf	*Euphorbia orientalis* L.	IDTDA26
Tindouf	*Euphorbia orientalis* L.	IDTDA27
Tindouf	*Echinops* spp.	IDTDA28
Tindouf	*Echinops* spp.	IDTDA29
Tindouf	*Echinops* spp.	IDTDA30
Tindouf	*Euphorbia bupleuroides*	IDTDA31
Tindouf	*Euphorbia bupleuroides*	IDTDA32
Tindouf	*Euphorbia bupleuroides*	IDTDA33
Tindouf	*Peganum harmala*	IDTDA34
Tindouf	*Peganum harmala*	IDTDA35
Tindouf	*Peganum harmala*	IDTDA36
**Morocco**		
**City**	**Botanical Species**	**ID**
Khénifra	*Ziziphus christi*	IDKM37
Khénifra	*Ziziphus christi*	IDKM38
Khénifra	*Ziziphus christi*	IDKM39
Khénifra	*Multifloral*	IDKM40
Khénifra	*Multifloral*	IDKM41
Khénifra	*Multifloral*	IDKM42
Khénifra	*Citrus sinensis*	IDKM43
Khénifra	*Citrus sinensis*	IDKM44
Khénifra	*Citrus sinensis*	IDKM45
Khénifra	*Euphorbia orientalis* L.	IDKM46
Khénifra	*Euphorbia orientalis* L.	IDKM47
Khénifra	*Euphorbia orientalis* L.	IDKM48
Beni Mellal	*Citrus sinensis*	IDBM49
Beni Mellal	*Citrus sinensis*	IDBM50
Beni Mellal	*Citrus sinensis*	IDBM51
Beni Mellal	*Euphorbia orientalis* L.	IDBM52
Beni Mellal	*Euphorbia orientalis* L.	IDBM53
Beni Mellal	*Euphorbia orientalis* L.	IDBM54
Beni Mellal	*Ziziphus lotus*	IDBM55
Beni Mellal	*Ziziphus lotus*	IDBM56
Beni Mellal	*Ziziphus lotus*	IDBM57
Beni Mellal	*Eucalyptus globulus*	IDBM58
Beni Mellal	*Eucalyptus globulus*	IDBM59
Beni Mellal	*Eucalyptus globulus*	IDBM60
Azilal	*Thymus* spp.	IDAM61
Azilal	*Thymus* spp.	IDAM62
Azilal	*Thymus* spp.	IDAM63
Azilal	*Ziziphus lotus*	IDAM64
Azilal	*Ziziphus lotus*	IDAM65
Azilal	*Ziziphus lotus*	IDAM66
Azilal	*Multifloral*	IDAM67
Azilal	*Multifloral*	IDAM68
Azilal	*Multifloral*	IDAM69
Azilal	*Euphorbia orientalis* L.	IDAM70
Azilal	*Euphorbia orientalis* L.	IDAM71
Azilal	*Euphorbia orientalis* L.	IDAM72
Fquih Ben Salah	*Multifloral*	IDFM73
Fquih Ben Salah	*Multifloral*	IDFM74
Fquih Ben Salah	*Multifloral*	IDFM75
Fquih Ben Salah	*Eucalyptus globulus*	IDFM76
Fquih Ben Salah	*Eucalyptus globulus*	IDFM77
Fquih Ben Salah	*Eucalyptus globulus*	IDFM78
Fquih Ben Salah	*Globularia alypum*	IDFM79
Fquih Ben Salah	*Globularia alypum*	IDFM80
Fquih Ben Salah	*Globularia alypum*	IDFM81
Fquih Ben Salah	*Euphorbia orientalis* L.	IDFM82
Fquih Ben Salah	*Euphorbia orientalis* L.	IDFM83
Fquih Ben Salah	*Euphorbia orientalis* L.	IDFM84

**Table 2:** Time and gradient program of chromatographic analysis.

Time (min)	Solvent B (%)	Solvent A (%)
0.01	55	45
7.00	65	35
14.00	70	30
20.00	70	30
23.00	90	10
25.00	100	0
35.00	100	0

**Table 3:** Analytical validation of the HPLC-FLD method for biogenic amine determination.

Analytes	R^2^	LOD (mg/Kg)	LOQ (mg/Kg)	Recovery (%)	Precision (RSD%)	
					Intraday	Interday
Cadaverine	0.9988	0.003	0.01	94.50 ± 2.75	1.1	1.2
Histamine	0.9980	0.003	0.01	95.00 ± 2.88	1.2	1.3
Serotonin	0.9983	0.015	0.05	97.20 ± 1.45	1.0	1.1
Putrescine	0.9986	0.003	0.01	92.75 ± 0.80	1.2	1.3
Tryptamine	0.9983	0.015	0.05	95.50 ± 0.75	1.0	1.1
Tyramine	0.9987	0.003	0.01	93.10 ± 0.85	1.1	1.2
Spermidine	0.9995	0.003	0.01	95.50 ± 1.20	1.2	1.3
Spermine	0.9980	0.003	0.01	91.65 ± 0.65	1.1	1.3

**Table 4:** Worst-case Estimated Daily Intake (EDI) of individual biogenic amines from selected Algerian and Moroccan honeys under different consumption scenarios.

EDI Worst Case						
	Adult—Medium Consumption	Adult—High Consumption	Child—Medium Consumption	Child—High Consumption	Botanical Species	Country
**EDI Serotonin** **(mg/kg bw/day)**	0.000135	0.000337	0.000189	0.000566	*Ziziphus lotus*	A
	0.000294	0.000736	0.000412	0.001237	*Multifloral*	M
**EDI Tryptamine** **(mg/kg bw/day)**	0.000169	0.000423	0.000237	0.000710	*Euphorbia orientalis* L.	A
	0.000210	0.000525	0.000294	0.000881	*Thymus* spp.	M
**EDI Cadaverine** **(mg/kg bw/day)**	0.000040	0.000100	0.000056	0.000168	*Tamarix* L.	A
	0.000042	0.000106	0.000059	0.000178	*Multifloral*	M
**EDI Histamine** **(mg/kg bw/day)**	0.000058	0.000146	0.000082	0.000245	*Euphorbia orientalis* L.	A
	0.000059	0.000147	0.000083	0.000248	*Euphorbia orientalis* L.	M
**EDI Tyramine** **(mg/kg bw/day)**	0.000730	0.001825	0.001022	0.003067	*Euphorbia orientalis* L.	A
	0.000028	0.000070	0.000039	0.000117	*Multifloral*	M
**EDI Putrescine** **(mg/kg bw/day)**	0.000031	0.000076	0.000043	0.000128	*Euphorbia orientalis* L.	A
	0.000028	0.000069	0.000039	0.000116	*Multifloral*	M
**EDI Spermidine** **(mg/kg bw/day)**	0.000071	0.000178	0.000100	0.000299	*Euphorbia bupleuroides*	A
	0.000026	0.000066	0.000037	0.000111	*Multifloral*	M
**EDI Spermine** **(mg/kg bw/day)**	0.000085	0.000213	0.000119	0.000358	*Bunium mauritanicum*	A
	0.000076	0.000190	0.000106	0.000319	*Multifloral*	M

Estimated Daily Intake (EDI, mg/kg bw/day) calculated for adults and children under medium and high honey consumption scenarios. Values represent worst-case exposure conditions based on the highest detected concentrations for each botanical species. Honeys were classified according to botanical origin, and country of production is indicated as A (Algeria) and M (Morocco).

**Table 5:** Total biogenic amines (TBA), Biogenic Amine Index (BAI), and Quality Index (QI) in Algerian and Moroccan honeys according to botanical origin.

Algeria			
Botanical Species	Total Biogenic Amines (TBA; mg/kg)	Biogenic Amine Index (BAI)	Quality Index (QI)
*Eucalyptus globulus*	0.348	0.082	0.082
*Ziziphus lotus*	0.783	0.167	0.167
*Euphorbia orientalis* L.	3.005	2.800	0.244
*Tamarix* L.	1.068	0.333	0.220
*Multifloral*	1.075	0.373	0.237
*Citrus sinensis*	1.245	0.271	0.204
*Echinops* spp.	1.377	0.308	0.230
*Bunium mauritanicum*	1.336	0.480	0.201
*Euphorbia orientalis* L.	1.179	0.260	0.260
*Echinops* spp.	1.585	0.309	0.111
*Euphorbia bupleuroides*	1.214	0.165	0.117
*Peganum harmala*	1.311	0.207	0.065
**Morocco**			
**Botanical Species**	**Total Biogenic Amines (TBA; mg/kg)**	**Biogenic Amine Index (BAI)**	**Quality Index (QI)**
*Ziziphus christi*	1.324	0.286	0.172
*Multifloral*	1.046	0.349	0.218
*Citrus sinensis*	0.673	0.148	0.107
*Euphorbia orientalis* L.	1.077	0.232	0.232
*Citrus sinensis*	0.673	0.148	0.107
*Euphorbia orientalis* L.	0.694	0.078	0.063
*Ziziphus lotus*	0.917	0.185	0.153
*Eucalyptus globulus*	0.918	0.190	0.169
*Thymus* spp.	0.896	0.064	0.064
*Ziziphus lotus*	0.960	0.251	0.203
*Multifloral*	1.231	0.361	0.315
*Euphorbia orientalis* L.	1.273	0.327	0.270
*Multifloral*	2.348	0.417	0.235
*Eucalyptus globulus*	0.612	0.152	0.110
*Globularia alypum*	0.993	0.266	0.223
*Euphorbia orientalis* L.	1.064	0.355	0.321

**Table 6:** Vasoactive Amine Load (VAL) and Potentiation Index (PI) in Algerian and Moroccan honeys according to botanical origin and country of production.

Algeria		
Botanical Species	Vasoactive Amine Load (VAL; mg/kg)	Potentiation Index (PI)
*Eucalyptus globulus*	0.044	0.038
*Ziziphus lotus*	0.046	0.122
*Euphorbia orientalis* L.	2.635	0.165
*Tamarix* L.	0.100	0.233
*Multifloral*	0.174	0.199
*Citrus sinensis*	0.136	0.134
*Echinops* spp.	0.126	0.182
*Bunium mauritanicum*	0.342	0.138
*Euphorbia orientalis* L.	0.204	0.056
*Echinops* spp.	0.270	0.039
*Euphorbia bupleuroides*	0.086	0.079
*Peganum harmala*	0.175	0.032
**Morocco**		
**Botanical Species**	**VAL (mg/kg)**	**PI**
*Ziziphus christi*	0.253	0.032
*Multifloral*	0.173	0.176
*Citrus sinensis*	0.102	0.046
*Euphorbia orientalis* L.	0.188	0.044
*Citrus sinensis*	0.102	0.046
*Euphorbia orientalis* L.	0.033	0.045
*Ziziphus lotus*	0.098	0.084
*Eucalyptus globulus*	0.108	0.086
*Thymus* spp.	0.001	0.064
*Ziziphus lotus*	0.139	0.113
*Multifloral*	0.184	0.188
*Euphorbia orientalis* L.	0.250	0.079
*Multifloral*	0.220	0.197
*Eucalyptus globulus*	0.074	0.072
*Globularia alypum*	0.140	0.126
*Euphorbia orientalis* L.	0.242	0.104

## Data Availability

The original contributions presented in this study are included in the article/Supplementary Material. Further inquiries can be directed to the corresponding author.

## Supplementary Materials

The following supporting information can be downloaded at: https://www.mdpi.com/article/10.3390/foods15081411/s1, Figure S1: Concentrations of serotonin, tryptamine and cadaverine in honey samples from Algeria and Morocco. Differences between the same botanical species and the same city (a: sample 1 vs. sample 2; b: sample 1 vs. sample 3; c: sample 2 vs. sample 3; *p* < 0.05). Data are expressed as the mean ± standard deviation; Figure S2: Concentrations of histamine, tyramine and putrescine in honey samples from Algeria and Morocco. Differences between the same botanical species and the same city (a: sample 1 vs. sample 2; b: sample 1 vs. sample 3; c: sample 2 vs. sample 3; *p* < 0.05). Data are expressed as the mean ± standard deviation.; Figure S3: Concentrations of spermidine and spermine in honey samples from Algeria and Morocco. Differences between the same botanical species and the same city (a: sample 1 vs. sample 2; b: sample 1 vs. sample 3; c: sample 2 vs. sample 3; *p* < 0.05). Data are expressed as the mean ± standard deviation; Figure S4: Concentrations of biogenic amines in *E. globus* honey samples from Algeria and Morocco. Significant differences compared to IDLA1 ^a^; Significant differences compared to IDLA2 ^b^; Significant differences compared to IDLA3 ^c^; Significant differences compared to IDBM58 ^d^; Significant differences compared to IDBM59 ^e^; Significant differences compared to IDBM60 ^f^; *p* < 0.05. Data are expressed as the mean ± standard deviation; Figure S5: Concentrations of biogenic amines in *Z. lotus* honey samples from Algeria and Morocco. Significant differences compared to IDLA4 ^a^; Significant differences compared to IDLA5 ^b^; Significant differences compared to IDLA6 ^c^; Significant differences compared to IDBM56 ^d^; *p* < 0.05. Data are expressed as the mean ± standard deviation; Figure S6: Concentrations of biogenic amines in *E. orientalis* honey samples from Algeria and Morocco. Significant differences compared to IDLA7 ^a^; Significant differences compared to IDLA8 ^b^; Significant differences compared to IDLA9 ^c^; Significant differences compared to IDTDA25 ^d^; Significant differences compared to IDTDA26 ^e^; Significant differences compared to IDTDA27 ^f^; Significant differences compared to IDKM46 ^g^; Significant differences compared to IDKM47 ^h^; Significant differences compared to IDKM48 ^i^; Significant differences compared to IDBM52 ^j^; Significant differences compared to IDBM53 ^k^; Significant differences compared to IDBM54 ^l^; Significant differences compared to IDAM70 ^m^; Significant differences compared to IDAM71 ^n^; *p* < 0.05. Data are expressed as the mean ± standard deviation; Figure S7: Concentrations of biogenic amines in multifloral honey samples from Algeria and Morocco. Significant differences compared to IDTA13 ^a^; Significant differences compared to IDTA14 ^b^; Significant differences compared to IDTA15 ^c^; Significant differences compared to IDKM40 ^d^; Significant differences compared to IDKM41 ^e^; Significant differences compared to IDKM42 ^f^; Significant differences compared to IDAM67 ^g^; Significant differences compared to IDAM68 ^h^; Significant differences compared to IDAM69 ^i^; *p* < 0.0001. Data are expressed as the mean ± standard deviation; Figure S8: Concentrations of biogenic amines in *C. sinensis* honey samples from Algeria and Morocco. Significant differences compared to IDTA16 ^a^; Significant differences compared to IDTA17 ^b^; Significant differences compared to IDTA18 ^c^; *p* < 0.05. Data are expressed as the mean ± standard deviation; Figure S9: Pearson correlation analysis among biogenic amine concentrations; between biogenic amine concentrations and total estimated daily intake (EDI) in Algeria (A) and Morocco (M). Correlation coefficients (r) indicate the strength and direction of linear associations, with statistical significance set at *p* < 0.05. Positive correlations show an increase in amine concentrations or exposure estimates; negative correlations indicate inverse relationships.
