# Supplementary material for: Toxicological Relevance of Biogenic Amines in Honey: Dietary Exposure and Integrated Risk Indicators in Algerian and Moroccan Honeys
Source: Foods. 2026 Apr 17;15(8):1411. doi: 10.3390/foods15081411 (PMC13115006; doi:10.3390/foods15081411)
Supplement: Supplementary file 1 [file foods-15-01411-s001.zip › foods-4222268-supplementary.pdf]

foods

MDPI

Supplementary Materials

Intra-City Assessment of Biogenic Amine Content in Honeys of Identical Botanical Origin

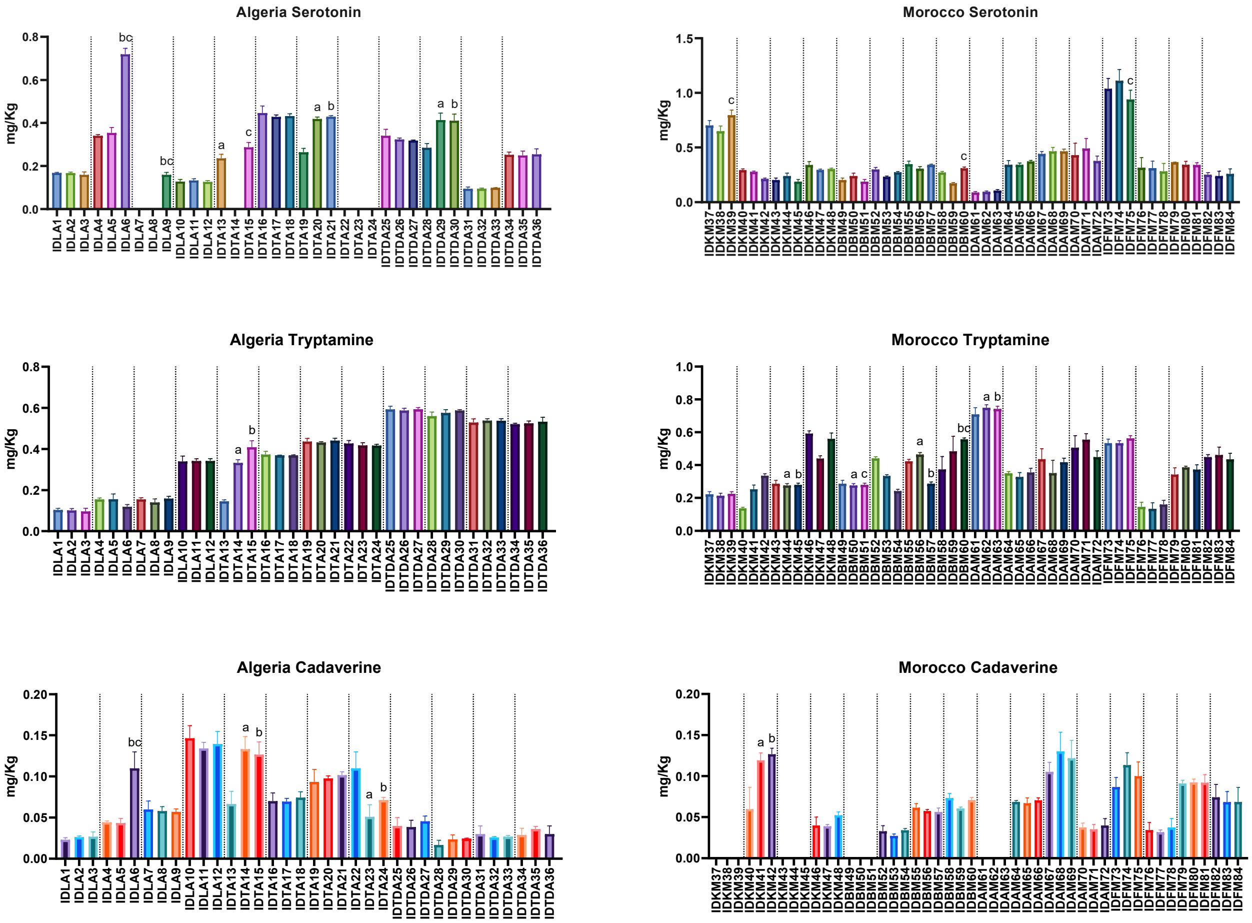

Figure S1. Concentrations of serotonin, tryptamine and cadaverine in honey samples from Algeria and Morocco. Differences between the same botanical species and the same city (a: sample 1 vs sample 2; b: sample 1 vs sample 3; c: sample 2 vs sample 3; $p < 0.05$). Data are expressed as the mean ± standard deviation.

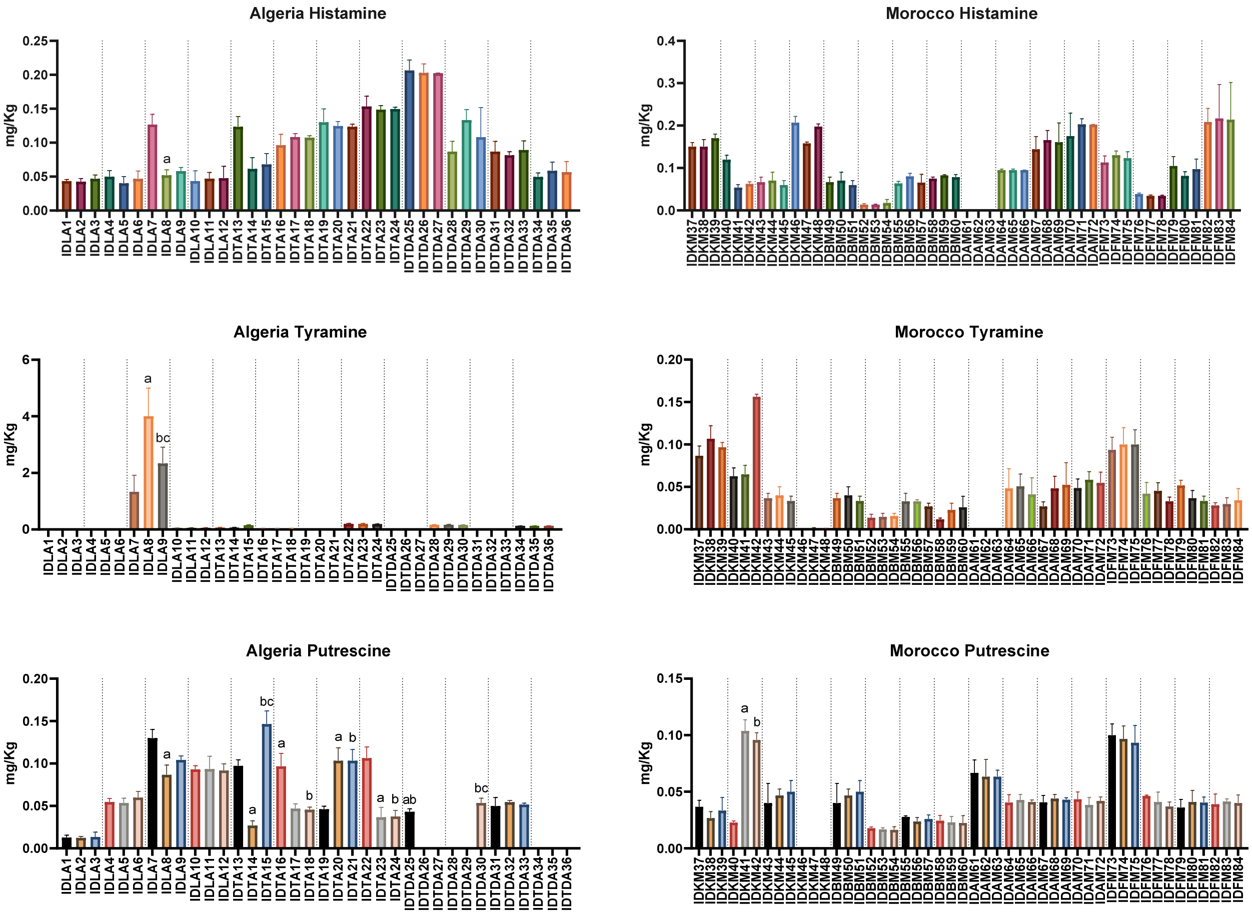

Figure S2. Concentrations of histamine, tyramine and putrescine in honey samples from Algeria and Morocco. Differences between the same botanical species and the same city (a: sample 1 vs sample 2; b: sample 1 vs sample 3; c: sample 2 vs sample 3; $p < 0.05$). Data are expressed as the mean ± standard deviation.

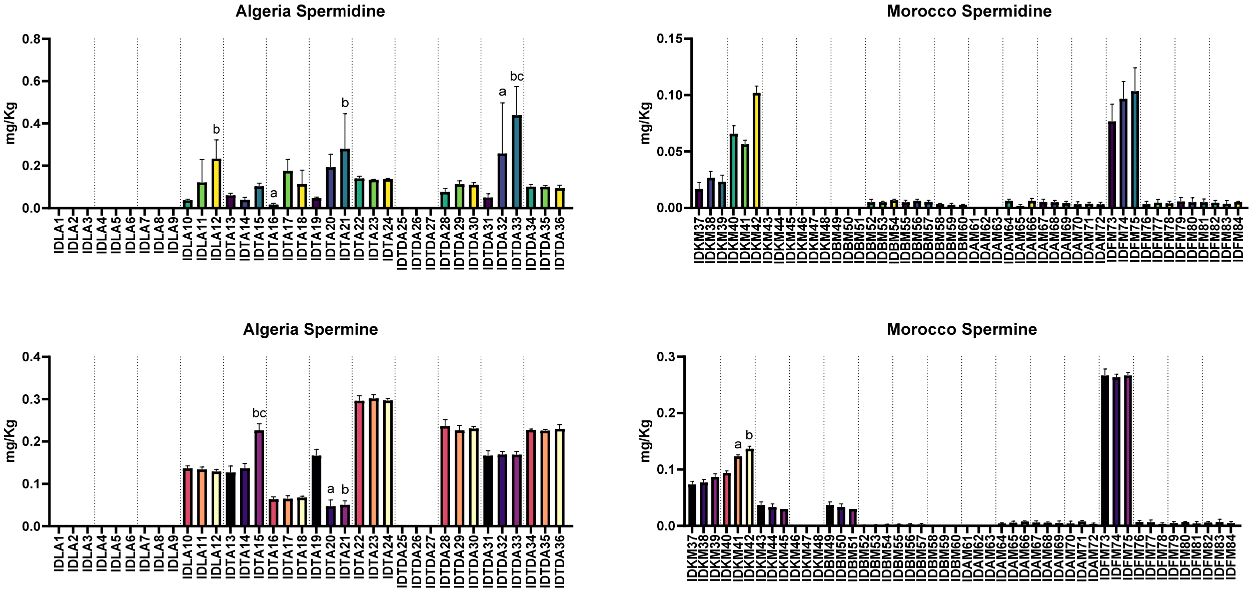

Figure S3. Concentrations of spermidine and spermine in honey samples from Algeria and Morocco. Differences between the same

botanical species and the same city (a: sample 1 vs sample 2; b: sample 1 vs sample 3; c: sample 2 vs sample 3; $p < 0.05$). Data are expressed as the mean ± standard deviation

Inter-City Variability of Biogenic Amines in Honeys of the Same Botanical Origin

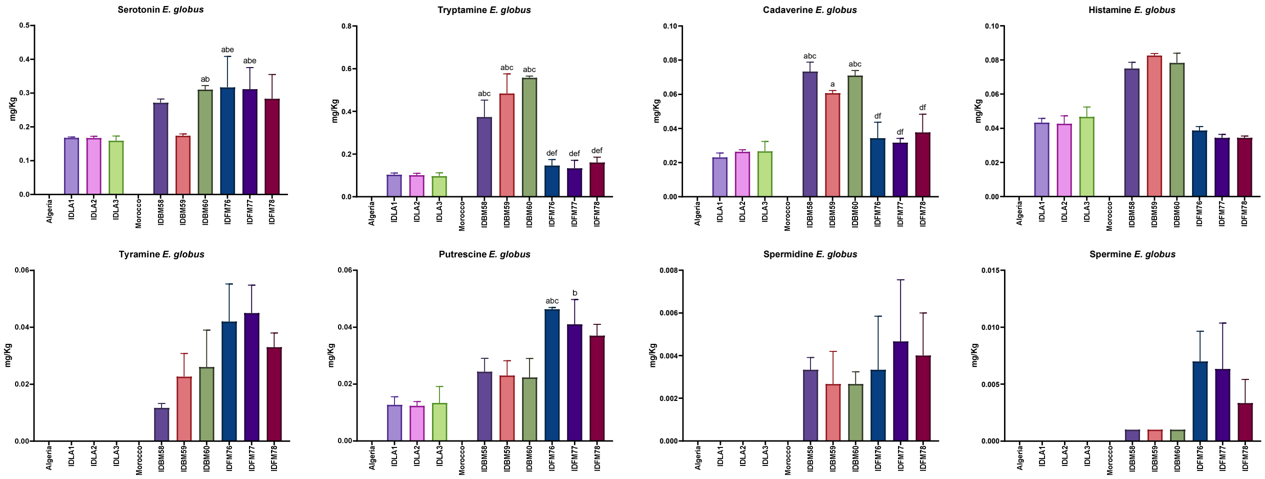

Figure S4. Concentrations of biogenic amines in *E. globus* honey samples from Algeria and Morocco. Significant differences compared to IDLA1 [a]; Significant differences compared to IDLA2 [b]; Significant differences compared to IDLA3 [c]; Significant differences compared to IDBM58 [d]; Significant differences compared to IDBM59 [e]; Significant differences compared to IDBM60 [f]; $p < 0.05$. Data are expressed as the mean ± standard deviation.

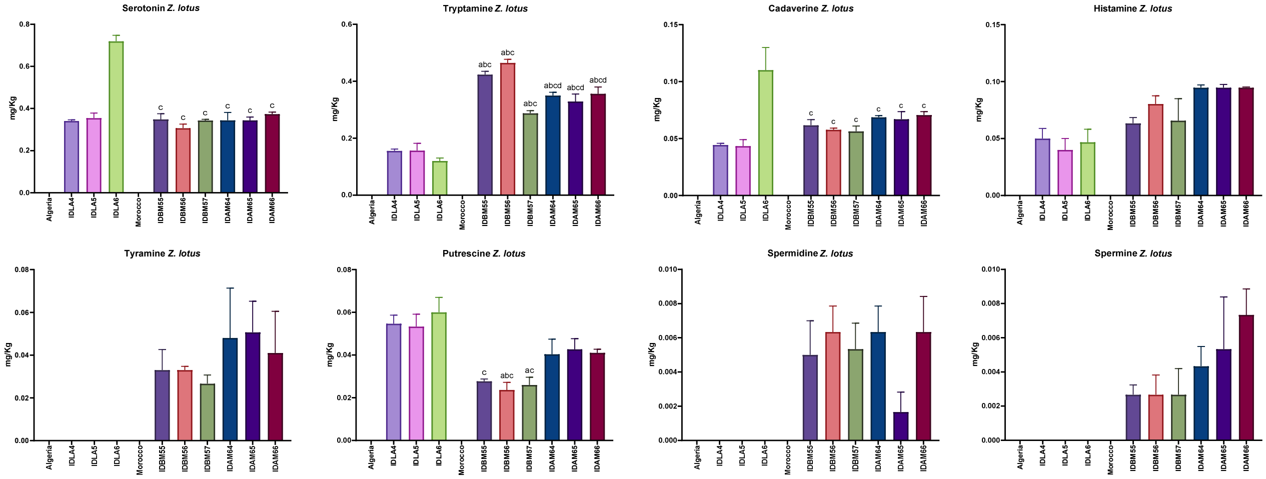

Figure S5. Concentrations of biogenic amines in *Z. lotus* honey samples from Algeria and Morocco. Significant differences compared to IDLA4 [a]; Significant differences compared to IDLA5 [b]; Significant differences compared to IDLA6 [c]; Significant differences compared to IDBM56 [d]; $p < 0.05$. Data are expressed as the mean ± standard deviation.

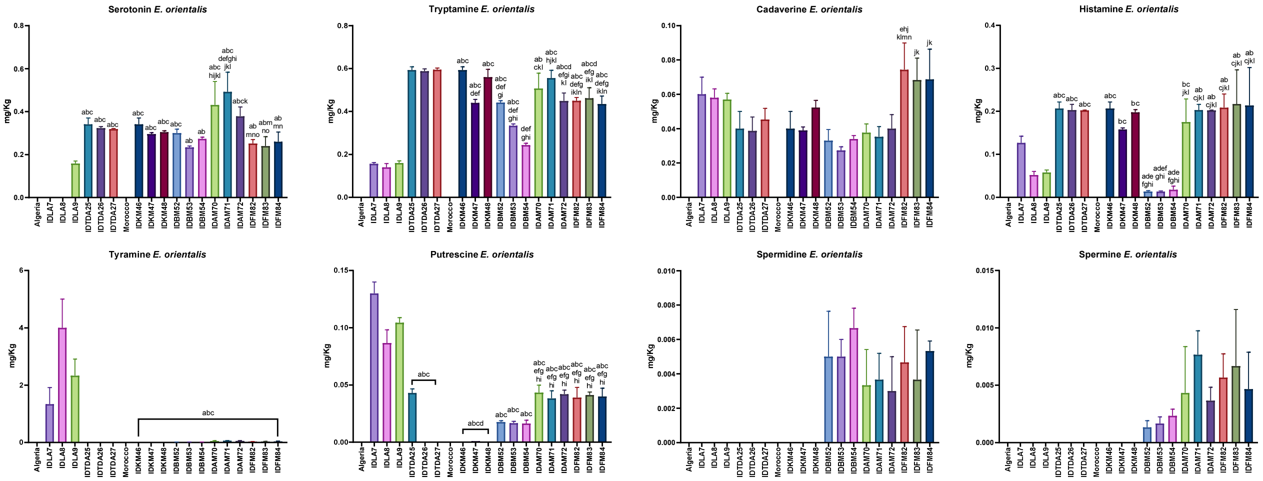

Figure S6. Concentrations of biogenic amines in *E. orientalis* honey samples from Algeria and Morocco. Significant differences compared to IDLA7 [a]; Significant differences compared to IDLA8 [b]; Significant differences compared to IDLA9 [c]; Significant differences compared to IDTDA25 [d]; Significant differences compared to IDTDA26 [e]; Significant differences compared to IDTDA27 [f]; Significant differences compared to IDKM46 [g]; Significant differences compared to IDKM47 [h]; Significant differences compared to IDKM48 [i]; Significant differences compared to IDBM52 [j]; Significant differences compared to IDBM53 [k]; Significant differences compared to IDBM54 [l]; Significant differences compared to IDAM70 [m]; Significant differences compared to IDAM71 [n]; $p < 0.05$. Data are expressed as the mean ± standard deviation.

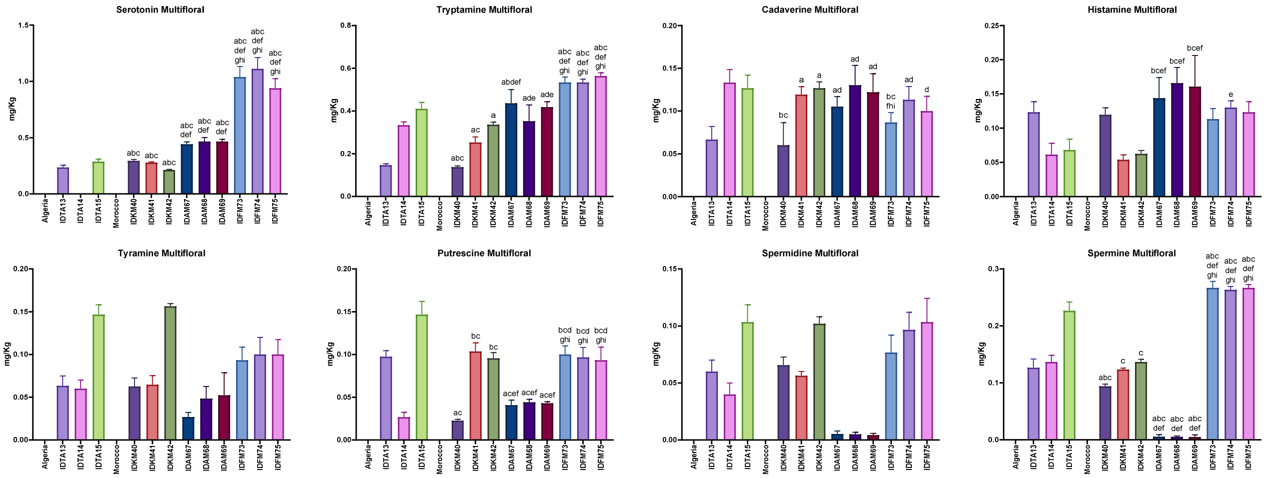

Figure S7. Concentrations of biogenic amines in multifloral honey samples from Algeria and Morocco. Significant differences compared to IDTA13 [a]; Significant differences compared to IDTA14 [b]; Significant differences compared to IDTA15 [c]; Significant differences compared to IDKM40 [d]; Significant differences compared to IDKM41 [e]; Significant differences compared to IDKM42 [f]; Significant differences compared to IDAM67 [g]; Significant differences compared to IDAM68 [h]; Significant differences compared to IDAM69 [i]; $p < 0.0001$. Data are expressed as the mean ± standard deviation.

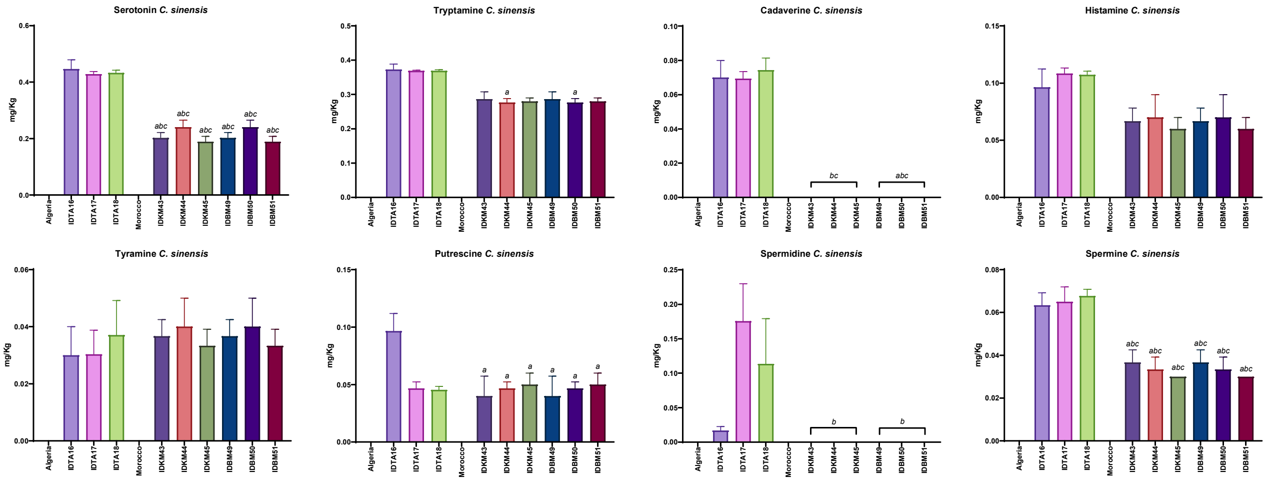

Figure S8. Concentrations of biogenic amines in *C. sinensis* honey samples from Algeria and Morocco. Significant differences compared to IDTA16 [a]; Significant differences compared to IDTA17 [b]; Significant differences compared to IDTA18 [c]; $p < 0.05$. Data are expressed as the mean ± standard deviation.

Correlations among Biogenic Amines, Exposure Scenarios and Total EDI in Algeria and Morocco

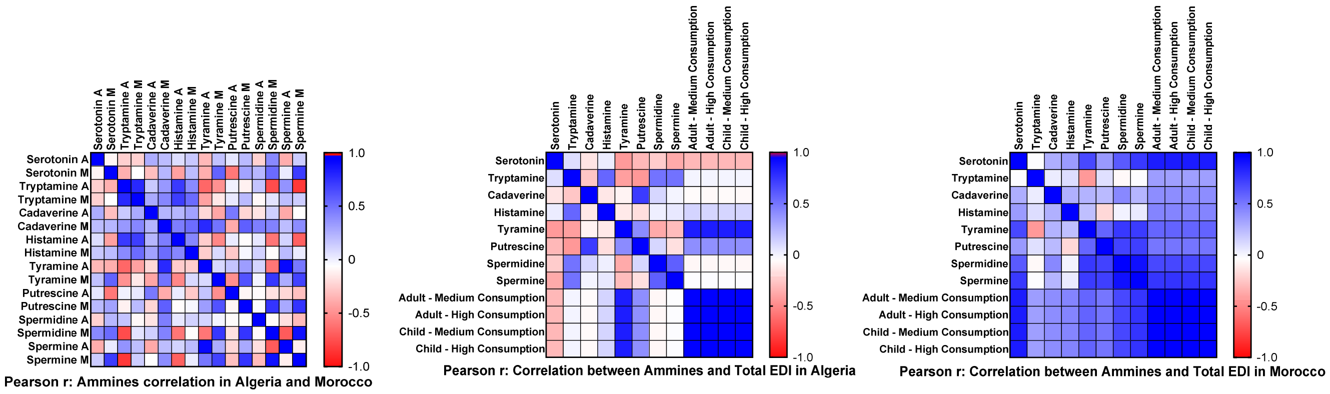

Figure S9. Pearson correlation analysis among biogenic amine concentrations; between biogenic amine concentrations and total estimated daily intake (EDI) in Algeria (A) and Morocco (M). Correlation coefficients (r) indicate the strength and direction of linear associations, with statistical significance setted at $p < 0.05$. Positive correlations show an increase in amine concentrations or exposure estimates, negative correlations indicate inverse relationships.
